# Uterine Vulnerability to Environmental PM_2.5_: Chronic Wood Smoke Exposure Alters Morphogenesis Before First Pregnancy

**DOI:** 10.3390/ijms27104289

**Published:** 2026-05-12

**Authors:** Francisca Villarroel, Eder Ramírez, Nikol Ponce, Francisco Nualart, Felipe Ramírez-Cepeda, Luis Mercado, Maria Angélica Miglino, Paulo Salinas

**Affiliations:** 1 Laboratory of Animal & Experimental Morphology, Institute of Biology, Faculty of Sciences, Pontificia Universidad Católica de Valparaíso, Valparaíso 2362807, Chile; 2 Laboratory of Neurobiology and Stem Cells NeuroCellT, Department of Cellular Biology, Faculty of Biological Sciences, Universidad de Concepción, Concepción 4070386, Chile; 3 PhD Program in Morphological Sciences, Universidad de La Frontera, Temuco 4811230, Chile; 4 Center of Excellence in Surgical and Morphological Studies (CEMyQ), Universidad de La Frontera, Temuco 4811230, Chile; 5 Center for Advanced Microscopy CMA BIO-BIO, Universidad de Concepción, Concepción 4070386, Chile; 6 Laboratorio de Inmunología, Institute of Biology, Faculty of Sciences, Pontificia Universidad Católica de Valparaíso, Valparaíso 2362807, Chile; 7 Department of Veterinary Medicine, Universidade de Marília (UNIMAR), Marília 17525-902, São Paulo, Brazil

**Keywords:** wood smoke air pollution, environmental reproductive toxicology, uterine remodeling, transgenerational exposure, hypoxia, inflammation

## Abstract

Chronic exposure to fine particulate matter (${\mathrm{PM}}_{2.5}$) derived from residential wood combustion is a major environmental health concern in southern Chile and other cold-climate regions. Although ${\mathrm{PM}}_{2.5}$ has been linked to adverse reproductive outcomes, it remains unclear whether sustained exposure induces pregestational uterine alterations that compromise reproductive competence before the first pregnancy. This study evaluated the effects of chronic wood smoke-derived ${\mathrm{PM}}_{2.5}$ exposure on uterine morphology and molecular markers in nulliparous rats. A two-generation exposure model was used to assess cumulative effects. Second-generation (G2) female Sprague Dawley rats continuously exposed from conception were housed in filtered air (FA, control; n=12) or ${\mathrm{PM}}_{2.5}$-containing ambient air (NFA; n=12) until reproductive maturity (82 days). Uterine horns were analyzed by histology, planimetry, immunohistochemistry, immunofluorescence, and second harmonic generation microscopy. Markers of hypoxia, inflammation, extracellular matrix remodeling, angiogenesis, proliferation, apoptosis, and DNA repair were quantified. Chronic ${\mathrm{PM}}_{2.5}$ exposure increased hypoxia-inducible factor 1α, tumor necrosis factor-α, vascular endothelial growth factor A, and collagen types I, III, and IV, while transforming growth factor-β expression and Ki-67-positive proliferating cells were reduced. Exposed rats showed increased apoptosis and decreased nuclear expression of $O^{6}$-methylguanine-DNA methyltransferase, indicating impaired DNA repair capacity. Second harmonic generation imaging demonstrated increased collagen deposition with marked fibrillar disorganization. These findings indicate that chronic wood smoke-derived ${\mathrm{PM}}_{2.5}$ exposure induces hypoxia-driven structural and molecular alterations in the uterus of nulliparous rats before first pregnancy, including extracellular matrix remodeling, inflammatory imbalance, angiogenic dysregulation, reduced proliferation, and compromised DNA repair, suggesting early disruption of uterine homeostasis and increased susceptibility to adverse reproductive outcomes.

## 1. Introduction

Air pollution is currently one of the most pressing global public health challenges [1,2]. Among regulated pollutants, fine particulate matter ≤ 2.5 µm (${\mathrm{PM}}_{2.5}$) is considered the most harmful due to its capacity to penetrate the respiratory system, translocate into the systemic circulation, and exert toxic effects in distal organs. From a particle toxicology perspective, ${\mathrm{PM}}_{2.5}$ represents a complex mixture whose physicochemical properties, size, and combustion origin critically determine biological outcomes across multiple organ systems. In southern Chile and other cold-climate regions, a major source of ${\mathrm{PM}}_{2.5}$ is residential wood biomass combustion [3,4]. During winter, mean ${\mathrm{PM}}_{2.5}$ concentrations in Temuco range between 40 and 60 µg/m^3^, with peaks exceeding 120–150 µg/m^3^ [5,6], surpassing by more than threefold the WHO 24-h guideline of 15 µg/m^3^ [1] and resulting in chronic environmental exposure. The mean concentration used in our exposure chambers (∼45 µg/m^3^) closely replicates these real-world levels, enabling evaluation of toxicological effects under environmentally relevant conditions [6,7]. Such exposure is particularly hazardous due to the presence of carbonaceous particles, polycyclic aromatic hydrocarbons, and volatile compounds capable of inducing oxidative injury even at low concentrations [8,9,10,11,12].

Epidemiological and experimental evidence has demonstrated that ${\mathrm{PM}}_{2.5}$ exposure elicits both acute and chronic effects on multiple organs and tissues throughout the life cycle [13,14,15,16,17,18,19,20,21,22]. Beyond respiratory and cardiovascular systems, growing evidence indicates that reproductive organs are vulnerable targets of particulate matter toxicity, although the underlying mechanisms and critical windows of susceptibility remain incompletely understood. At the molecular level, one of the key mechanisms involves disruption of oxygen metabolism and overexpression of hypoxia-inducible factor 1-α (HIF-1α) [23,24,25]. HIF-1α activation initiates cascades of inflammation, cytotoxicity, and oxidative stress, leading to structural damage and compromising the integrity of epithelial and vascular barriers [17,26,27,28,29]. In parallel, infertility rates have steadily increased and currently affect approximately 17% of the global population [1]. Chronic exposure to ${\mathrm{PM}}_{2.5}$ has emerged as a significant environmental factor associated with disruptions in ovarian function, hormonal dysregulation, and earlier onset of menopause [30,31,32]. From a toxicological standpoint, these reproductive effects have been linked to persistent inflammation, vascular remodeling, fibrotic responses, and impaired cellular homeostasis in reproductive tissues [33], as well as alterations in DNA repair with potential implications for both fetal and adult health [34,35].

Our experimental findings show that gestational exposure to wood smoke-derived ${\mathrm{PM}}_{2.5}$ reduces placental oxygen diffusion and compromises fetal development by sustaining a persistent hypoxic state and concomitantly impairing placental glucose uptake through alterations in glucose transporter expression and distribution, thereby limiting the combined oxygen–nutrient supply to the fetus [6,7,36]. Similarly, in nulliparous rats chronically exposed to ${\mathrm{PM}}_{2.5}$, increased collagen deposition and thickening of the uterine wall have been observed [37]. Our group also reported that this exposure does not alter serum concentrations of estradiol or progesterone in nulliparous rats [6,37], suggesting that ${\mathrm{PM}}_{2.5}$-induced uterine alterations occur independently of systemic ovarian–hypothalamic–pituitary axis disruption and are instead driven by local tissue-level toxicological mechanisms. We have also shown that wood smoke-derived ${\mathrm{PM}}_{2.5}$ is chemically heterogeneous and enriched in carbonaceous material, mineral components, and redox-active metals, supporting its intrinsic capacity to induce oxidative stress, hypoxia, and inflammatory signaling at the tissue level [6]. Related findings indicate that ${\mathrm{PM}}_{2.5}$ exposure alters uterine architecture and angiogenesis during gestation [7]. However, despite increasing evidence of adverse pregnancy outcomes associated with ${\mathrm{PM}}_{2.5}$ exposure, it remains largely unknown whether chronic environmental exposure programs uterine tissue vulnerability before the first pregnancy, thereby predisposing the uterus to maladaptive responses during gestation. Together, these findings support the hypothesis that placental effects observed during pregnancy may originate from preexisting uterine alterations induced by early and sustained ${\mathrm{PM}}_{2.5}$ exposure, generating a hypoxic, pro-inflammatory, and structurally remodeled microenvironment even before conception.

We hypothesize that sustained multigenerational exposure to wood smoke-derived ${\mathrm{PM}}_{2.5}$ induces hypoxia-driven toxicological alterations in the uterus of nulliparous rats, leading to persistent changes in extracellular matrix organization, angiogenic signaling, cellular turnover, and DNA repair capacity that may accumulate across developmental windows prior to the first pregnancy. The aim of this study was to evaluate the effects of continuous transgenerational ${\mathrm{PM}}_{2.5}$ exposure—spanning prenatal, postnatal, and juvenile stages—on uterine morphology of second-generation (G2) nulliparous rats before reproductive onset. Morphological, cellular, and molecular alterations were assessed using histology, immunohistochemistry, immunofluorescence, and second harmonic generation microscopy to identify early toxicological targets and integrated tissue-level responses to environmentally relevant multigenerational ${\mathrm{PM}}_{2.5}$ exposure. This work provides preclinical evidence supporting the concept that the uterus is a sensitive target organ of particulate matter toxicity across generations and highlights the importance of considering cumulative exposure burdens during critical developmental windows, with potential implications for human reproductive health in regions with persistent air pollution.

## 2. Results

### 2.1. Air Pollution Exposure

Second-generation (G2) female rats experienced continuous ${\mathrm{PM}}_{2.5}$ exposure from conception through postnatal day 82. The daily average pollutant concentrations at the exposure site were 48.8 µg/m^3^ (±36.1; CV = 74%; ${\mathrm{CI}}_{95\%}$ = 42–56) for ${\mathrm{PM}}_{2.5}$, 56.9 µg/m^3^ (±38.3; CV = 67.3%; ${\mathrm{CI}}_{95\%}$ = 48.6–65.2) for PM_10_, and 0.78 ppm (±0.49; CV = 61.5%) for CO (Supplementary Document S3). In the NFA chamber, ${\mathrm{PM}}_{2.5}$ averaged 44.6 µg/m^3^ (±9.8; CV = 21.9%; ${\mathrm{CI}}_{95\%}$ = 42.2–47.0), closely matching ambient levels, whereas in the FA chamber it was significantly reduced to 3.0 µg/m^3^ (±1.3; CV = 34.3%; *p* < 0.001), achieving a 94% reduction. These chamber concentrations were sustained under the assigned exposure conditions throughout the experimental timeline, thereby reflecting chronic environmentally relevant exposure for the NFA group and near-background particulate levels for the FA group. To provide an approximate indicator of exposure magnitude, we estimated the inhaled ${\mathrm{PM}}_{2.5}$ mass using the mean chamber concentration and a standardized ventilation assumption. The total inhaled air volume per female was calculated as 0.041 L min^−1^ over a 22-day reference window (1299 L; 1.299 m^3^). Based on these parameters, the estimated inhaled ${\mathrm{PM}}_{2.5}$ mass during this reference period was 57.9 µg in the NFA chamber (44.6 µg/m^3^ × 1.299 m^3^) and 3.9 µg in the FA chamber (3.0 µg/m^3^ × 1.299 m^3^). These calculations are intended solely as an order-of-magnitude comparison between exposure conditions and do not represent deposited or retained dose, as particle deposition fractions, clearance dynamics, and inter-individual variability in minute ventilation were not modeled. ${\mathrm{PM}}_{2.5}$ concentration curves during the study period are presented in Supplementary Document S4.

### 2.2. Uterine Response to Hypoxia: Tissue Markers and Structural Integrity

#### 2.2.1. Hypoxia (HIF-1α)

**Figure 1:**
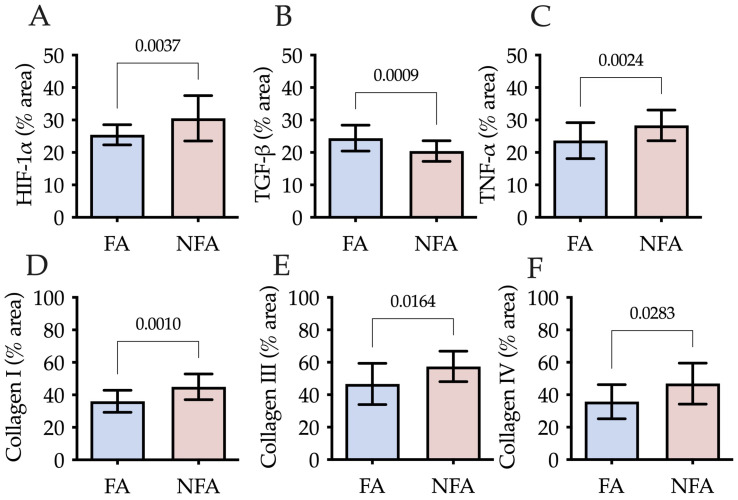
Uterine tissue markers of hypoxia, inflammation, and extracellular matrix remodeling in nulliparous rats chronically exposed to fine particulate matter (${\mathrm{PM}}_{2.5}$). Comparative analysis between rats exposed to filtered air (FA) and non-filtered air (NFA). (**A**) Hypoxia-inducible factor 1-alpha (HIF-1α, % stained area); (**B**) Transforming growth factor beta (TGF-β, % stained area); (**C**) Tumor necrosis factor alpha (TNF-α, % stained area); (**D**) Collagen type I (% stained area); (**E**) Collagen type III (% stained area); (**F**) Collagen type IV (% stained area). Data are presented as mean ± SD. *p* values are indicated for statistically significant differences between groups (*p* < 0.05; unpaired Student’s *t*-test).

**Figure 2:**
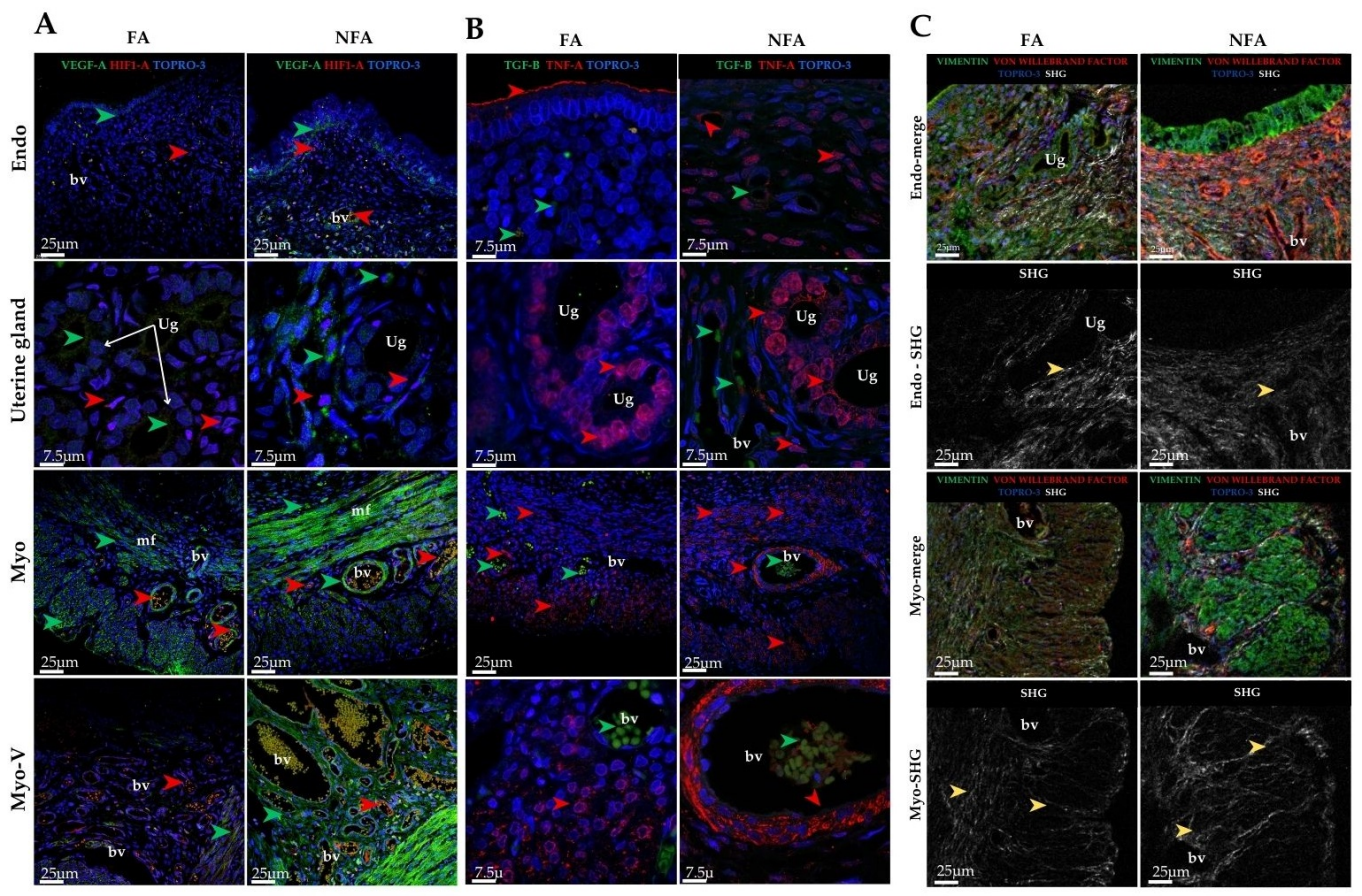
Assessment of inflammatory, angiogenic, and structural markers in the uterine horn of nulliparous rats chronically exposed to ${\mathrm{PM}}_{2.5}$ using immunofluorescence and second harmonic generation (SHG) microscopy. Representative transverse sections from uterine horns of adult nulliparous Sprague–Dawley rats (82 days old) exposed from birth to reproductive maturity to filtered air (FA) or non-filtered air (NFA). (**A**) Immunofluorescence for vascular endothelial growth factor A (VEGF-A; green) and hypoxia-inducible factor 1-alpha (HIF-1α; red), with To-pro3 nuclear counterstaining (blue). Representative images correspond to the endometrium (Endo), uterine glands (Ug), myometrium (Myo), and vascular layer of the myometrium (Myo-V). VEGF-A-positive and HIF-1α-positive cells are indicated by green and red arrows, respectively. (**B**) Immunofluorescence for transforming growth factor beta (TGF-β; green) and tumor necrosis factor alpha (TNF-α; red), with To-pro3 nuclear counterstaining (blue). Representative images show labeling in the deep endometrial layer (Endo), uterine glands (Ug), myometrium (Myo), and vascular layer of the myometrium (Myo-V). TGF-β-positive cells are indicated by green arrows, whereas TNF-α-positive cells are indicated by red arrows. (**C**) Second harmonic generation (SHG; white) microscopy combined with immunofluorescence for vimentin (green), von Willebrand factor (red), and To-pro3 (blue). ${\mathrm{PM}}_{2.5}$ exposure in the NFA group was associated with reduced collagen organization in both the endometrium (Endo) and myometrium (Myo) compared with FA animals. Disorganized collagen fibers are indicated by yellow arrows. Blood vessels (Bv) and smooth muscle fibers (mf) are indicated for anatomical reference.

Quantification of HIF-1α by area fraction revealed statistically significant differences between exposure groups (p=0.0037), with higher expression observed in the ${\mathrm{PM}}_{2.5}$-exposed group (NFA) compared with the control group (FA) (Figure 1A). In the endometrium, positive staining was more intense in the deep layer, particularly near the superficial epithelium. At the level of the uterine glands, both groups showed positive staining; however, this signal was clearly evident around the glands only in the NFA group. Blood vessels in the endometrium and broad ligament exhibited HIF-1α positivity in both groups. In the myometrium, staining was detected in the circular layer in both groups, whereas it was markedly higher in the longitudinal layer of the NFA group (Figure 2A).

#### 2.2.2. Inflammation

Quantification of TGF-β and TNF-α in uterine tissue revealed significant differences between the exposure groups. Compared to the control group (FA), rats exposed to ${\mathrm{PM}}_{2.5}$ (NFA) showed a lower area fraction of TGF-β (*p* = 0.0009; Figure 1B) and a higher fraction of TNF-α (*p* = 0.0024; Figure 1C). Although differences were subtle at the qualitative level, quantitative threshold analysis confirmed significant group differences in TGF-β and TNF-α labeling (Figure 1B,C). TGF-β was localized in the superficial epithelium, uterine glands, and endometrial lymphocytes, with more intense staining in the FA group. Furthermore, TGF-β exhibited intraluminal vascular staining in some endometrial vessels. The signal appeared to be associated with luminal cellular elements; however, given the absence of nucleated erythrocytes and the known biology of TGF-β, this pattern is interpreted cautiously as vascular-associated staining rather than definitive erythrocyte expression. In the myometrium, both groups displayed similar staining patterns across the circular, vascular, and longitudinal layers. TNF-α immunoreactivity was predominantly cytoplasmic, with occasional perinuclear accumulation patterns observed in epithelial and stromal cells. No definitive intranuclear localization was identified. This distribution pattern is consistent with TNF-α intracellular trafficking and receptor-associated signaling processes rather than direct nuclear localization. Increased staining intensity was evident in the NFA group, particularly in the basal endometrial layer and around uterine glands. In the myometrium, staining was detected within longitudinal smooth muscle fibers, with higher expression in the exposed group (Figure 2B).

#### 2.2.3. Collagen Remodeling and Fibrillar Disorganization

Immunohistochemical analysis revealed a significant increase in the area fraction of COL I (*p* = 0.0171; Figure 1D), COL III (*p* = 0.0213; Figure 1E), and COL IV (*p* = 0.0283; Figure 1F) in NFA compared to FA. COL I immunoreactivity was predominantly observed within the endometrial extracellular matrix and in subepithelial stromal regions adjacent to the superficial epithelium, with greater staining in the deep endometrial layer of the exposed group. In the myometrium, increased staining was detected within interstitial connective tissue of both the circular and longitudinal layers in NFA. COL III showed a similar distribution pattern within stromal compartments, perivascular connective tissue, and gland-associated extracellular matrix. Although intense labeling was observed along the epithelial–stromal interface in both groups, the NFA group exhibited increased positivity in the deep endometrial stroma and around uterine glands. In the myometrium, staining was concentrated around vascular structures and interstitial connective tissue, with greater intensity in NFA. COL IV immunoreactivity was localized primarily along basement membrane structures underlying the superficial epithelium and surrounding uterine glands and vascular endothelium. Increased basement membrane-associated staining was observed in the NFA group. In the myometrium, COL IV labeling was detected along vascular basement membranes and connective tissue interfaces across layers, with more pronounced intensity in ${\mathrm{PM}}_{2.5}$-exposed animals (Supplementary Document S5). SHG microscopy enabled the detection of total collagen content in uterine tissue and assessment of fiber organization. In both groups, collagen was primarily concentrated in the endometrium, surrounding the uterine glands and within the compact layer. However, in the ${\mathrm{PM}}_{2.5}$-exposed group (NFA), collagen was also detected in the deep endometrial layer, close to the superficial epithelium. In the myometrium, the highest proportion of collagen was located in the vascular and longitudinal layers, with greater abundance in NFA compared to the control group (FA). Regarding fiber orientation, collagen in the FA group exhibited a parallel arrangement, whereas in the NFA group, despite an overall increase in collagen content, fibers appeared disorganized (Figure 2C).

### 2.3. Epithelial Receptivity and HB-EGF Expression

**Figure 3:**
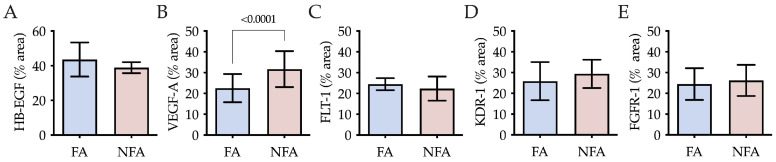
Uterine expression of growth factors and angiogenic markers in nulliparous rats chronically exposed to ${\mathrm{PM}}_{2.5}$. Comparison between filtered air (FA) and non-filtered air (NFA) exposure groups. Immunohistochemical quantification of the stained area (%) for: (**A**) Heparin-binding EGF-like growth factor (HB-EGF), (**B**) Vascular endothelial growth factor A (VEGF-A), (**C**) Fms-related tyrosine kinase 1 (FLT-1), (**D**) Kinase insert domain receptor 1 (KDR-1), and (**E**) Fibroblast growth factor receptor 1 (FGFR1). Data are presented as mean ± SD. Statistically significant differences between groups are indicated (*p* < 0.05; unpaired Student’s *t*-test).

Quantification of HB-EGF revealed no statistically significant differences between exposure groups (*p* = 0.1275); however, the ${\mathrm{PM}}_{2.5}$-exposed group (NFA) showed a lower mean area fraction compared to the control group (FA) (Figure 3A). Regarding the integrity of the endometrial surface, both groups exhibited similar epithelial organization. Nevertheless, in the NFA group, the superficial epithelium appeared slightly more fibrillar in morphology, whereas a cuboidal arrangement predominated in the FA group (Supplementary Document S6).

### 2.4. Expression of Angiogenic Factors

Area fraction quantification revealed a significant increase in VEGF-A expression in the ${\mathrm{PM}}_{2.5}$-exposed group (NFA) compared to the control group (FA) (*p* < 0.0001) (Figure 3B), while no statistically significant differences were observed for Flt-1 (*p* = 0.0891) (Figure 3C), Kdr-1 (*p* = 0.1524) (Figure 3D), or FGFR-1 (*p* = 0.5133) (Figure 3E). VEGF-A was localized in the superficial epithelium, uterine glands, and stromal cells of the endometrium, with greater staining in the NFA group, particularly in the compact layer and around the glands. In the myometrium, staining was similar between groups and distributed across the circular, vascular, and longitudinal layers (Figure 2A). The increase in VEGF-A area fraction in NFA was mainly observed in endometrial compartments, whereas myometrial staining remained comparable between groups. Flt-1 and Kdr-1 receptors were expressed in the superficial epithelium, glands, and endometrial stroma in both groups. However, Flt-1 showed more intense staining in the superficial epithelium of the FA group, while Kdr-1 was more intensely stained in the deep endometrial layer of FA compared to NFA. In the myometrium, both receptors were localized in muscle fibers and blood vessels, with no marked differences between groups. FGFR-1 was also expressed in the superficial epithelium (predominantly in the apical region), glands, and endometrial stroma. In the myometrium, positive staining was observed in all layers, with greater intensity in the longitudinal layer of the NFA group compared to FA (Supplementary Document S7).

### 2.5. Cell Proliferation and Apoptosis

**Figure 4:**
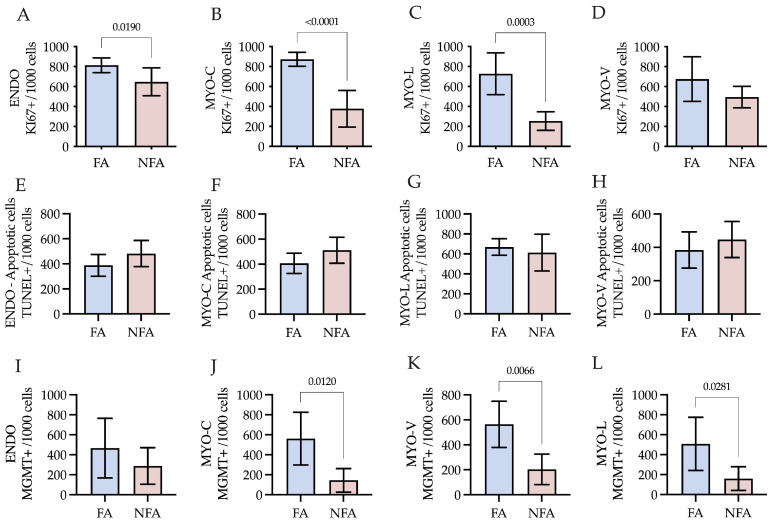
Effects of chronic ${\mathrm{PM}}_{2.5}$ exposure on cell proliferation (Ki-67), apoptosis (TUNEL), and DNA repair capacity (MGMT) in different uterine strata of nulliparous rats. Quantification of positive cells per 1000 total cells in uterine tissue from rats exposed to filtered air (FA) or non-filtered air (NFA). (**A**–**D**) Proliferating cells expressing Ki-67: (**A**) endometrium (ENDO), (**B**) circular muscle layer (MYO-C), (**C**) longitudinal muscle layer (MYO-L), and (**D**) vascular layer (MYO-V). (**E**–**H**) Apoptotic cells (TUNEL^+^): (**E**) ENDO, (**F**) MYO-C, (**G**) MYO-L, and (**H**) MYO-V. (**I**–**L**) DNA repair marker MGMT: (**I**) ENDO, (**J**) MYO-C, (**K**) MYO-V, and (**L**) MYO-L. Bars represent mean ± SD. Significant differences between groups are indicated (*p* < 0.05; unpaired Student’s *t*-test).

**Figure 5:**
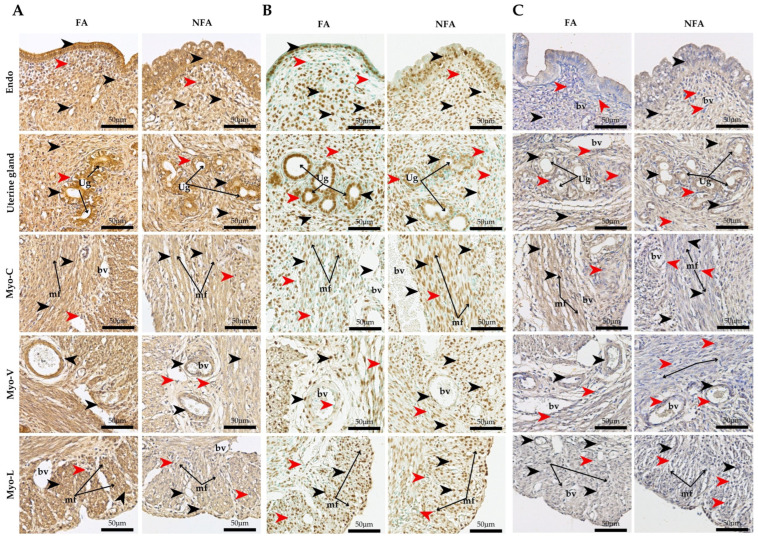
Detection of proliferation, apoptosis and DNA methylation in the uterine horn of nulliparous rats exposed to ${\mathrm{PM}}_{2.5}$ (FA vs. NFA). Representative transverse sections of the uterine horn from adult nulliparous Sprague Dawley rats (82 days old) exposed to filtered air (FA) or non-filtered air (NFA). Panel (**A**). Immunohistochemistry for the proliferation marker Ki-67. ${\mathrm{PM}}_{2.5}$ exposure resulted in a decreased number of Ki-67-positive cells across multiple uterine layers. Positive (black arrows) and negative (red arrows) cells are indicated in: deep endometrial layer (Endo), Ug, compact endometrium (Endo), Myo-C, Myo-V, and Myo-L. Bv and mf are also identified. Panel (**B**). TUNEL assay for apoptotic cell detection. ${\mathrm{PM}}_{2.5}$ exposure was associated with an overall increase in the number of TUNEL^+^ cells; however, no significant differences were observed across individual uterine layers. Positive cells (black arrows) and negative cells (red arrows) are shown in: (**A**,**B**) deep endometrial layer (Endo), uterine glands (Ug), compact endometrium (Endo), circular muscle layer (Myo-C), vascular layer (Myo-V), and longitudinal muscle layer (Myo-L). Blood vessels (Bv); smooth muscle fibers (mf). Panel (**C**). Immunohistochemistry for the global DNA methylation marker MGMT. Chronic ${\mathrm{PM}}_{2.5}$ exposure was associated with a reduced number of MGMT-positive cells, particularly in myometrial layers. Distribution of positive (black arrows) and negative (red arrows) cells is shown in: Endo, Ug, Myo-C, Myo-V, and Myo-L. Bv and mf are highlighted.

The rate of cell proliferation, assessed by counting Ki-67-positive cells, was significantly lower in the ${\mathrm{PM}}_{2.5}$-exposed group (NFA) compared to the control group (FA) (*p* = 0.0004). Stratified analysis revealed significant differences in the endometrium (*p* = 0.0190) (Figure 4A) and in all three layers of the myometrium: circular (*p* = 0.0012) (Figure 4B), vascular (*p* = 0.0223) (Figure 4C), and longitudinal (*p* = 0.0003) (Figure 4D), with a consistent reduction in NFA (Figure 5A). Apoptotic activity, evaluated by the number of TUNEL^+^ cells, did not differ significantly between exposure groups when considering the uterus as a whole (*p* = 0.3420). Likewise, no statistically significant differences were detected at the compartmental level, either in the endometrium (*p* = 0.1548; Figure 4E) or in the myometrium, including the circular (*p* = 0.1020; Figure 4F), vascular (*p* = 0.3608; Figure 4G), and longitudinal layers (*p* = 0.5471; Figure 4H). Nevertheless, the ${\mathrm{PM}}_{2.5}$-exposed group (NFA) exhibited a higher mean number of TUNEL^+^ cells compared to the control group (FA), although this difference did not reach statistical significance (Figure 5B).

### 2.6. Alteration in DNA Repair Capacity Due to ${\mathrm{PM}}_{2.5}$ Exposure

DNA repair capacity, estimated by nuclear positivity for MGMT, was significantly lower in ${\mathrm{PM}}_{2.5}$-exposed rats (NFA) compared to the control group (FA) (*p* = 0.0306). No statistically significant differences were detected in the endometrium between the groups (*p* = 0.2870) (Figure 4I). Stratified analysis revealed marked reductions in the myometrium: circular (*p* = 0.0128) (Figure 4J), vascular (*p* = 0.0067) (Figure 4K) and longitudinal (*p* = 0.0279; Figure 4L) layers.

Supplementary Document S8 contains the complete quantitative analysis of uterine markers associated with hypoxia, inflammation, ECM remodeling, angiogenesis, cell proliferation, apoptosis, and DNA repair in nulliparous rats exposed to filtered air (FA) or non-filtered air (NFA).

## 3. Discussion

The use of G2 animals with continuous exposure across two generations represents a model of multigenerational (as opposed to strictly transgenerational) exposure, where effects may accumulate through direct exposure of germ cells and developing tissues. This design is particularly relevant for environmental pollutants like ${\mathrm{PM}}_{2.5}$, where human populations often experience sustained exposure across generations in polluted regions. While not designed to isolate epigenetic inheritance mechanisms, this model captures the integrated biological burden of prolonged environmental insult. The exposure system was strategically located in Temuco, Chile, where the experimental chambers were continuously supplied with ambient urban air representative of a pollution scenario dominated by biomass combustion. The non-filtered air (NFA) chamber therefore reflected the actual environmental ${\mathrm{PM}}_{2.5}$ concentrations characteristic of this urban area, whereas the filtered air (FA) chamber incorporated a high-efficiency particulate air filtration system that reduced ${\mathrm{PM}}_{2.5}$ levels to approximately 3.0 µg/m^3^, representing an overall reduction of approximately 94% relative to the ambient-exposed NFA chamber. This experimental configuration enabled direct comparison between environmentally relevant chronic exposure conditions and near-background particulate levels.

Under these environmentally realistic conditions, the present study demonstrates that chronic exposure to wood smoke-derived ${\mathrm{PM}}_{2.5}$ is associated with the development of a hypoxic, inflammatory, and pro-fibrotic uterine microenvironment in nulliparous rats. These changes are consistent with a coordinated response involving hypoxia-driven signaling, inflammatory activation, and extracellular matrix remodeling, rather than isolated molecular changes. Similar uterine and systemic alterations have been reported following exposure to ${\mathrm{PM}}_{2.5}$ from traffic-related and industrial sources, which are also known to induce oxidative stress, endothelial dysfunction, and collagen remodeling [38,39,40]. In the present model, the marked overexpression of HIF-1α is consistent with sustained tissue hypoxia, whereas the concomitant up-regulation of VEGF-A suggests an adaptive angiogenic response that may be insufficient to fully restore tissue homeostasis.

At the inflammatory level, the increase in TNF-α observed in ${\mathrm{PM}}_{2.5}$-exposed animals aligns with previous reports linking chronic particulate exposure to persistent activation of pro-inflammatory cytokines and fibrotic remodeling pathways [41]. In contrast, the reduction in TGF-β expression suggests alterations in signaling pathways involved in tissue repair and homeostatic regulation [42]. No significant differences were detected in the expression of HB-EGF, an epithelial receptivity marker; however, the marked reduction in Ki-67-positive cells, together with a global, though not layer-specific, increase in apoptosis as assessed by TUNEL labeling, indicates an imbalance between cellular proliferation and programmed cell death. Reduced MGMT expression likely reflects compromised DNA repair capacity associated with sustained hypoxia and inflammatory stress, consistent with previous observations in hypoxic reproductive tissues [43], rather than representing evidence of a direct epigenetic modification. Although several of these differences reached statistical significance, their magnitude was generally moderate, suggesting that the observed phenotype likely represents an early stage of tissue remodeling rather than overt functional impairment. Taken together, these findings indicate that chronic exposure to wood smoke-derived ${\mathrm{PM}}_{2.5}$ may initiate early morphological and molecular alterations in the uterus prior to the first pregnancy. Accordingly, these changes should be interpreted as indicative of altered uterine microenvironmental regulation, rather than direct evidence of impaired reproductive function.Nevertheless, any implications for fertility or reproductive outcomes remain speculative and require confirmation through longitudinal functional and reproductive assessments.

### 3.1. Uterine Hypoxia and Inflammatory Response

Our results reveal a significant increase in HIF-1α expression in the ${\mathrm{PM}}_{2.5}$-exposed group, supporting the hypothesis that chronic particulate exposure is associated with the establishment of a hypoxic microenvironment in uterine tissue prior to the onset of gestation. Predominant localization of HIF-1α within the endometrial epithelial barrier and uterine glands indicates compensatory activation of the HIF-1α axis in response to oxygen reduction, consistent with physiological mechanisms observed across the estrous cycle [44]. Although this compensatory response may initially be adaptive, persistent HIF-1α activation under chronic exposure conditions may become maladaptive over time, favoring persistent inflammation and tissue damage [45,46,47,48].

The simultaneous decrease in TGF-β and increase in collagen accumulation in ${\mathrm{PM}}_{2.5}$-exposed animals (NFA) is consistent with a fibrotic-like remodeling process potentially involving noncanonical inflammatory pathways, particularly TNF-α activity. While TGF-β is recognized for its central role in fibrosis induction [49], its reduction here raises the possibility of alternative routes—potentially mediated by TNF-α-dependent mechanisms. This pro-inflammatory cytokine, also elevated in the exposed group, not only amplifies the production of other inflammatory mediators but also has been shown to stimulate fibroblast proliferation and extracellular matrix synthesis in other experimental contexts [50], contributing to the pro-fibrotic phenotype observed. Persistent inflammatory signaling in this context may reflect early alterations in uterine tissue homeostasis rather than direct evidence of functional impairment, as elevated endometrial TNF-α has been associated with reduced embryo viability and implantation defects during early development [51,52]. Accordingly, the sustained hypoxia and pro-fibrotic inflammation documented here are more appropriately interpreted as indicative of early-stage tissue remodeling processes, the functional consequences of which remain to be determined.

In some epithelial and glandular cells, TNF-α immunofluorescence exhibited partial perinuclear or apparent nuclear localization. This staining pattern is consistent with previously described intracellular trafficking and perinuclear signaling of TNF-α in endometrial tissue, where the cytokine and its receptors are abundantly expressed in glandular and stromal compartments throughout the estrous/menstrual cycle [53]. Such distribution has been associated with accumulation of membrane-bound pro-TNF and the internalization of TNF–TNFR1 complexes near the nucleus, a process that enables downstream activation of transcription factors such as NF-*κ*B and AP-1 [54]. In human endometrial stromal and epithelial cells, TNF-α stimulation induces nuclear translocation of NF-*κ*B and expression of IL-8, ICAM-1, and MMP-9, supporting the role of intracrine or perinuclear TNF signaling in regulating inflammatory and remodeling pathways [55]. Within the framework of this study, chronic exposure to wood smoke-derived ${\mathrm{PM}}_{2.5}$ may be associated with increased TNF-α-related intracellular signaling patterns in the uterine epithelium, mirroring mechanisms previously described in human endometrial inflammation and endometriosis. Accordingly, the apparent nuclear signal is interpreted as consistent with intracellular cytokine trafficking and signaling activity rather than nonspecific staining, supporting the presence of an early pro-inflammatory signaling state in the exposed group rather than a definitive pathological condition.

TGF-β immunoreactivity was occasionally observed within intraluminal vascular profiles in the endometrial microvasculature. Given that mature erythrocytes lack the cellular machinery required for cytokine synthesis, this staining pattern should be interpreted with caution. The intravascular signal may reflect circulating or platelet-derived TGF-β, adsorption to luminal components, or diffusion within plasma rather than active erythrocyte expression. Therefore, these vascular-associated signals were not considered indicative of erythrocyte production of TGF-β, but rather as part of the local vascular microenvironment under chronic ${\mathrm{PM}}_{2.5}$ exposure.

### 3.2. Extracellular Matrix Remodeling and Fibrosis

Chronic exposure to ${\mathrm{PM}}_{2.5}$ led to a significant increase in uterine collagen types I, III, and IV, supporting the activation of fibrogenic mechanisms within the ECM, most likely associated with sustained hypoxia and inflammatory signaling, and consistent with recent murine studies involving environmental toxicant exposure [56]. Consistent with these studies, our data support the notion that sustained hypoxia enhances collagen synthesis and secretion through post-transcriptional pathways [57,58]. In the uterus, the balance between ECM synthesis and degradation is essential for key reproductive processes such as decidualization, implantation, and vascular adaptation during gestation [59]. SHG microscopy revealed fibrillar disorganization in ${\mathrm{PM}}_{2.5}$-exposed animals (NFA), indicating altered fibrillogenesis compatible with the presence of activated myofibroblasts within a chronically inflamed microenvironment [60]. TNF-α overexpression, along with potential alterations in pathways related to relaxin-2 signaling, may negatively modulate matrix metalloproteinase activity, thereby promoting aberrant collagen accumulation [61,62]. Abnormal fibril orientation increases uterine stiffness [63], a structural change that has been associated in other biological contexts with altered tissue mechanics and impaired functional responses, including abnormal bleeding, dysmenorrhea, and implantation failure [64], as well as with dysregulation of genes involved in collagen synthesis and organization [63]. In the present study, however, the magnitude of collagen remodeling and fibrillar disorganization likely reflects an early-stage alteration in extracellular matrix organization rather than overt fibrotic pathology. These findings suggest that prolonged exposure to airborne pollutants such as ${\mathrm{PM}}_{2.5}$ may induce subclinical changes in uterine ECM architecture, which may influence tissue mechanical properties, although their direct functional impact on reproductive processes remains to be determined.

### 3.3. Endometrial Receptivity

Recent clinical evidence indicates that ${\mathrm{PM}}_{2.5}$ exposure prior to embryo transfer reduces the likelihood of clinical pregnancy by approximately 17% in in vitro fertilization cycles [65], highlighting the vulnerability of the implantation window to air pollution. While this evidence provides important clinical context, it should be interpreted cautiously in relation to the present experimental model, which does not directly assess implantation or pregnancy outcomes. To assess whether this pollutant affects uterine receptivity, we evaluated the expression of HB-EGF, a key mediator of decidualization and implantation [66]. Although no statistically significant differences were found between groups, a decreasing trend in HB-EGF expression was observed in the NFA group, which may reflect a subtle modulation of this factor under ${\mathrm{PM}}_{2.5}$-associated hypoxic conditions, without direct evidence of functional impairment. Importantly, the absence of statistically significant differences suggests that, under the conditions evaluated, chronic exposure alone may not be sufficient to alter basal HB-EGF expression in the non-pregnant uterus. The lack of significant differences suggests that chronic exposure alone may not be sufficient to impair basal HB-EGF expression in the absence of pregnancy. Nonetheless, previous studies have demonstrated that conditional loss of HB-EGF compromises the implantation window and reduces embryo implantation rates, thereby affecting reproductive efficiency [67]. However, these functional outcomes cannot be directly inferred from the present data, as HB-EGF expression remained largely unchanged. In line with this, we previously observed significantly smaller litters in rats exposed to ${\mathrm{PM}}_{2.5}$ during gestation [6]. These prior findings provide complementary evidence of reproductive vulnerability under exposure conditions, but they derive from a distinct experimental context involving gestational exposure and therefore should not be directly extrapolated to the present pre-gestational model. These findings suggest that while ${\mathrm{PM}}_{2.5}$ exposure may not directly interfere with implantation, it may influence uterine tissue programming processes that contribute to the quality of the peri-implantation environment. Accordingly, the observed changes are more consistent with subtle modulation of the uterine microenvironment rather than overt impairment of receptivity. These results support the hypothesis that chronic exposure to environmental pollutants may negatively impact the structural and molecular quality of the uterine environment, with potential implications for reproductive outcomes. However, the magnitude and functional significance of these alterations remain to be established in future studies incorporating direct reproductive endpoints.

### 3.4. Altered Angiogenesis Under Hypoxia

The overexpression of VEGF-A observed in uteri exposed to ${\mathrm{PM}}_{2.5}$ aligns with recent evidence indicating that this pollutant is associated with increased expression of components of the HIF-1α/VEGF-A axis in reproductive murine models [68] and supports the presence of a hypoxia-responsive angiogenic pathway in this experimental context. A lthough epidemiological studies have reported associations between ${\mathrm{PM}}_{2.5}$ exposure and adverse reproductive outcomes, such relationships should be interpreted cautiously in relation to the present model, which does not directly assess clinical endpoints [65]. Tissue hypoxia is, indeed, one of the most potent stimuli for VEGF induction and endometrial regeneration [69,70]. The increased VEGF-A expression in our NFA rats may be interpreted as an adaptive response aimed at restoring perfusion and preserving uterine homeostasis [71]. However, chronic overexpression of VEGF-A, when combined with sustained inflammation and epigenetic alterations, may shift from an adaptive response toward a dysregulated angiogenic profile. In the present study, the magnitude of VEGF-A up-regulation is consistent with an early-stage adaptive response rather than overt angiogenic dysfunction. Dysregulation of the angiogenic axis has been linked to leiomyomas and endometriosis, both characterized by aberrant angiogenesis and defective vascular maturation [72,73]. These associations, however, derive from pathological conditions and should not be directly extrapolated to the present findings. Moreover, ${\mathrm{PM}}_{2.5}$ exposure has been associated, in other experimental and epidemiological contexts, with epigenetic changes—such as hypermethylation of angiogenic gene promoters—that have been linked to increased susceptibility to these gynecological pathologies [74,75]. While such mechanisms provide a relevant biological framework, they were not directly assessed in this study and therefore remain speculative in this context. Although VEGF can support endometrial repair by promoting vascularization [76], in an inflamed and epigenetically dysregulated environment, such elevation may perpetuate an abnormal pro-angiogenic phenotype. In our model, however, these findings are more consistent with modulation of angiogenic signaling pathways rather than definitive evidence of pathological angiogenesis. In fact, the interplay between progesterone receptor B, VEGF and DNMT1 has been implicated in the pathogenesis of endometriosis, particularly in women with a history of low birth weight [77]. This latter observation is especially relevant, given that gestational exposure to ${\mathrm{PM}}_{2.5}$ reduced fetal weight [7], although these prior findings arise from a distinct experimental context and should not be directly extrapolated to the present pre-gestational model. Altogether, these findings suggest that ${\mathrm{PM}}_{2.5}$ exposure not only triggers a compensatory angiogenic response but also is associated with a uterine phenotype characterized by altered angiogenic profiles, which may reflect early microenvironmental adaptation to chronic exposure rather than established pathological remodeling, and whose functional consequences remain to be determined.

### 3.5. Alterations in Proliferation, Apoptosis and DNA Repair

Chronic ${\mathrm{PM}}_{2.5}$ exposure disrupted the balance between cell proliferation and death, as well as the genomic repair mechanisms in the uterus. We observed a significant reduction in Ki-67 expression in both the endometrium and myometrium, suggesting a decrease in cellular renewal capacity. Importantly, labeling rates represent the number of positive nuclei per 1000 segmented cells within each compartment and do not imply global tissue turnover. Although previous studies have associated ${\mathrm{PM}}_{2.5}$ exposure with oxidative stress, hypoxia, and cell cycle arrest through NOX4 activation and ROS generation [36,68,78,79], our results do not include direct measurements of oxidative stress markers or matrix remodeling enzymes. Therefore, while the observed reduction in proliferation and MGMT expression may reflect altered redox homeostasis, these mechanistic links remain hypothetical. Future studies should address ROS quantification and MMP/TIMP activity to confirm the oxidative and fibrogenic pathways inferred from the present morphological and immunohistochemical evidence. The lower TGF-β levels detected in our model may be compatible with this proposed mechanism, although further analyses are required to establish causality. In parallel, apoptotic activity (TUNEL^+^ cells) was increased, suggesting that the hypoxic–inflammatory microenvironment favors programmed cell death, potentially mediated by oxidative stress and imbalance in TGF-β signaling [80] However, given that these differences were modest and not consistently significant across compartments, they are more consistent with subtle shifts in cellular turnover rather than overt apoptotic dysregulation. The combined reduction in proliferation and increase in apoptosis may reflect an early alteration in the proliferative–apoptotic equilibrium, a process essential for successful implantation [34,81,82]. The reduction in MGMT expression observed in ${\mathrm{PM}}_{2.5}$-exposed animals indicates decreased DNA repair capacity within the uterine wall, consistent with reports of down-regulated repair enzymes following chronic particulate exposure. However, because the present study did not include direct analyses of promoter methylation or oxidative DNA adducts, these results should be interpreted as correlative evidence of impaired genomic maintenance rather than conclusive proof of epigenetic repression. Although promoter hypermethylation of DNA repair genes has been described after pollutant exposure [83], our findings are restricted to the morphological and immunohistochemical level. Future studies combining methylation assays or gene-expression profiling will be necessary to confirm whether MGMT modulation reflects true epigenetic regulation. Given that insufficient MGMT levels have been linked to low birth weight [84], and that we previously reported fetal growth restriction in rats exposed to wood smoke-derived ${\mathrm{PM}}_{2.5}$ [7], these observations provide a potential biological framework but should be interpreted cautiously, as they derive from different experimental contexts. Our findings—reduced Ki-67, increased apoptosis, and decreased MGMT—highlight that chronic ${\mathrm{PM}}_{2.5}$ exposure compromises uterine cellular homeostasis by diminishing its regenerative capacity and genomic integrity safeguards. In this context, these alterations are more consistent with early-stage disruption of tissue homeostasis rather than definitive evidence of functional impairment.

Apoptotic activity, assessed by TUNEL staining, showed a modest but consistent increase in the ${\mathrm{PM}}_{2.5}$-exposed group, although differences did not reach statistical significance at the compartmental level. Diffuse nuclear TUNEL labeling was observed in both groups, a pattern compatible with basal apoptotic turnover in uterine tissue; however, the higher mean number of TUNEL^+^ cells in NFA animals suggests a subtle shift toward increased programmed cell death under chronic hypoxic–inflammatory conditions. This finding aligns with evidence that sustained hypoxia and cytokine imbalance—particularly involving TGF-β and TNF-α—can promote apoptosis through oxidative and inflammatory pathways [85]. Taken together, reduced proliferation coupled with a tendency toward increased apoptosis reflects a disruption of the proliferative–apoptotic equilibrium, resulting in a quiescent but renewal-limited uterine environment. The magnitude of these changes suggests a modulation of cellular dynamics rather than a complete loss of regenerative capacity. Such imbalance is particularly relevant given that cyclical endometrial regeneration is essential for implantation competence and early pregnancy maintenance [34,81,82].

Chronic wood smoke ${\mathrm{PM}}_{2.5}$ exposure is associated with the establishment of an early hypoxic–inflammatory tissue imprint in the nulliparous uterus. We observed simultaneous up-regulation of HIF-1α and VEGF-A, echoing pre-clinical evidence that ${\mathrm{PM}}_{2.5}$ amplifies the HIF-1α/VEGF axis in reproductive tissues [68]. While this angiogenic surge may initially restore perfusion, it co-exists with sustained TNF-α-driven inflammation and reduced TGF-β, mirroring pro-fibrotic shifts seen in exposed endometrium [37]. Second-harmonic imaging confirmed disordered collagen I/III/IV architecture—an extracellular matrix phenotype linked to implantation failure and dysmenorrhea. However, these associations arise from pathological conditions and should not be directly extrapolated to the present findings. At the cellular level, ${\mathrm{PM}}_{2.5}$ curtailed Ki-67-defined proliferation and boosted TUNEL^+^ apoptosis, consistent with cellular responses previously associated with NOX4-related oxidative stress and cell-cycle regulation in other experimental models documented in neuronal and reproductive models [86,87]. The concomitant decline in MGMT aligns with mechanistic evidence showing that airborne pollutants can down-regulate DNA repair pathways, a process implicated in reproductive dysfunction and reduced female fertility, although direct causal links for MGMT in this context remain to be fully established. These uterine insults occurred without changes in systemic progesterone or estradiol, underscoring a direct effect independent of the gonadal axis. Although previous studies have reported associations between ${\mathrm{PM}}_{2.5}$ exposure and adverse reproductive outcomes, such as reduced litter size or altered pregnancy outcomes, these findings derive from different experimental or epidemiological contexts and should not be directly extrapolated to the present study. Collectively, the molecular and structural alterations observed here support the presence of an early uterine response to chronic ${\mathrm{PM}}_{2.5}$ exposure, characterized by hypoxia-associated signaling, inflammatory activation, and altered cellular turnover, the functional consequences of which remain to be determined.

### 3.6. Clinical Relevance and Implications for Human Reproductive Health

The results of this study suggest that chronic exposure to ${\mathrm{PM}}_{2.5}$ from wood combustion exerts a sustained and biologically relevant impact on uterine physiology by inducing an inflammatory and fibrogenic environment that interferes with essential processes such as angiogenesis, cellular proliferation and ECM organization. In the present experimental context, these alterations are more consistent with early-stage tissue remodeling rather than overt functional impairment. These changes carry potential clinical implications for women exposed to high levels of air pollution, particularly in regions where biomass is a primary source of heating. This type of exposure has been associated with reduced fertility and an increased risk of gestational complications such as preeclampsia and preterm birth, processes thought to be mediated by endothelial dysfunction, altered angiogenesis and chronic inflammatory imbalance [7,88,89]. However, these epidemiological associations should be interpreted cautiously in relation to the present findings, as this study does not directly evaluate reproductive or pregnancy outcomes. In this study, the increased VEGF-A expressions, collagen disorganization and reduced TGF-β observed in ${\mathrm{PM}}_{2.5}$-exposed uterine tissue reflect disruption of vascular and tissue remodeling mechanisms the structural and molecular level that could compromise uterine structural and vascular conditions that are required to support implantation and pregnancy progression [41]. Nevertheless, the magnitude of these alterations suggests modulation of tissue organization rather than definitive impairment of uterine function. Structurally, excessive or disorganized collagen deposition may reduce endometrial receptivity, while altered myometrial contractility may have implications for labor [33]. These potential functional consequences are inferred from previous studies and were not directly assessed in the present model. Furthermore, the persistence of a hypoxic and inflammatory uterine environment induced by chronic particulate exposure has been implicated, in other experimental and clinical contexts, in mechanisms associated with fibrogenic gynecological diseases such as uterine fibroids, endometriosis and postoperative adhesions [81]. Although these conditions share mechanistic features with the alterations observed here, direct extrapolation to pathological states should be made with caution. These conditions share common mechanisms with those observed in this experimental model, including oxidative stress, angiogenic dysfunction, and pathological tissue remodeling. Our findings support the concept that long-term exposure to wood smoke-derived ${\mathrm{PM}}_{2.5}$ may prime the uterus toward a maladaptive structural and molecular state, which may increase susceptibility to altered reproductive function, although direct evidence of dysfunction is beyond the scope of this study. Therefore, the results reinforce the need to recognize biomass-related air pollution as an emerging and underappreciated risk factor for female reproductive health, particularly in low- and middle-income regions where domestic wood combustion remains widespread. Future studies incorporating longitudinal and functional reproductive endpoints will be necessary to determine the clinical relevance of these findings.

### 3.7. Study Limitations

This study has limitations that should be considered when interpreting the findings. First, the lack of longitudinal follow-up prevents determining whether the uterine alterations persist or change during pregnancy or postpartum, limiting direct inference on fertility, implantation, and pregnancy maintenance; however, this gap was partly addressed by the inclusion of multiple molecular and cellular markers that characterize early exposure effects. Second, although the experimental design incorporated a two-generation exposure model to approximate cumulative environmental exposure, histological analyses were intentionally restricted to G2 females. While this approach increases ecological relevance by modeling sustained exposure across developmental stages, it does not allow definitive discrimination between cumulative multigenerational effects and single-generation adaptive responses. Therefore, the uterine phenotype observed in G2 should be interpreted as the integrated outcome of prolonged exposure rather than as evidence of a strictly transgenerational mechanism. Additionally, although estrous cyclicity was monitored and only females with regular cycles were included, tissue collection was not restricted to a specific estrous phase, and therefore residual cycle-related variability cannot be completely excluded. Third, mechanistic confirmation was limited by the absence of direct oxidative stress and fibrogenic activity measurements (e.g., ROS quantification, lipid peroxidation, and MMP/TIMP activity). Although increased HIF-1α and TNF-α expression and collagen disorganization by SHG are consistent with a hypoxic–pro-inflammatory and pro-fibrotic context, the proposed oxidative and remodeling pathways should be interpreted as inferential rather than causal. This limitation was partially mitigated by complementary immunohistochemistry for collagens I/III/IV and the evaluation of MGMT and TUNEL, which provide indirect insight into tissue remodeling and genomic stress. Fourth, because analyses were restricted to the uterus, we cannot distinguish local from systemic effects; future studies should incorporate other reproductive and neuroendocrine tissues (e.g., ovary, placenta, hypothalamus) and systemic inflammatory/endocrine biomarkers. Finally, while SHG enabled detailed visualization of collagen architecture, quantitative orientation metrics (e.g., coherency/alignment indices) were not computed and should be included in future work. Despite these limitations, internal validity was strengthened by stratified randomization, partial blinding, strict inclusion/exclusion criteria, and a power-informed sample size, together with standardized and validated histological, immunohistochemical and advanced imaging methods.

## 4. Materials and Methods

### 4.1. Exposure Site

The experimental procedures were conducted in Temuco, southern Chile (38°44^′^39.59^″^ S; 72°37^′^39.07^″^ W ), a city ranked as the sixth most polluted in the country [90]. Temuco represents a well-characterized environmental exposure scenario dominated by combustion-derived particulate matter, as residential wood-burning stoves have historically been the predominant source of airborne pollutants in this region [3,5,91,92,93]. The study was carried out during the austral winter, from June 15 to September 30, 2021, a period characterized by persistently high levels of ambient ${\mathrm{PM}}_{2.5}$ associated with residential heating demand. This seasonal window was intentionally selected to ensure sustained exposure to environmentally relevant concentrations of biomass-derived particles, consistent with real-world human exposure patterns. The study area did not present other significant industrial sources of atmospheric pollution, allowing attribution of airborne particulate exposure primarily to wood smoke combustion.

### 4.2. Exposure Conditions

**Figure 6:**
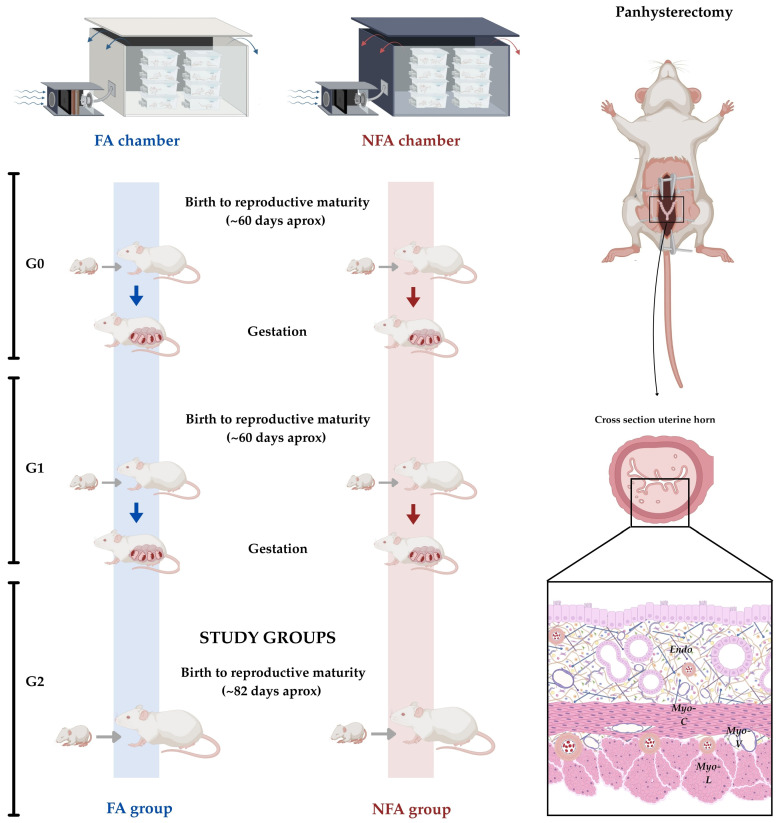
Two-generation exposure design and sampling of G2 animals. Founder females (G0) were exposed from birth to filtered air (FA) or wood smoke ${\mathrm{PM}}_{2.5}$ (NFA) and produced G1 offspring through mating with unexposed males. G1 females, maintained under continuous exposure, generated the G2 generation. Only G2 females were sampled at 82 days (dashed box), representing animals with integrated exposure spanning prenatal (as G1 fetuses), postnatal, and juvenile developmental windows. This design models cumulative environmental exposure across generations. Icons depict laparotomy and uterine sampling at the pregestational endpoint.

Two exposure chambers (adapted from [94]) were installed in the courtyard of the Faculty of Medicine at the University of La Frontera, in downtown Temuco, approximately 500 m from the local environmental air monitoring station (Supplementary Document S1; coordinates: −38.7496844990132, −72.6188400896599). This site is recognized for persistently high levels of ambient air pollution [95]. Each chamber measured 2.1 m × 2.0 m × 2.1 m and could accommodate up to 50 animal cages. Ambient air was continuously drawn directly from the external environment and introduced into both chambers at a rate of 20 m^3^ min^−1^ (flow rate 150 m^3^/h, 16.9 m^3^/h, 230 V; Zepol, S.L., model CBB60N, Barcelone, Spain ), ensuring that animals were exposed to the same air quality conditions as the local human population. Thus, rats housed in the non-filtered air (NFA) chamber inhaled the same mixture of particulate and gaseous pollutants present in Temuco’s atmosphere, representing true environmental exposure conditions rather than simulated concentrations. The system was normobaric, with internal pressure not exceeding 33 mmH_2_O. Both chambers operated under identical environmental parameters, but only one incorporated a multi-stage filtration system (Figure 6). This system included two initial filters (metal and pleated; 24 × 24 × 2 cm) to trap large- and medium-sized particles, a MERV8 particle filter, and a final HEPA PH97 filter removing 99.97% of particles >0.3 µm. Additionally, a Purafil PSA 102 device (500 cfm; Purafil Inc., Doraville, GA, USA) with Purafil Select medium in PK12 modules provided further air purification. All animals were maintained under standardized housing conditions: temperature 22 ± 2 °C, relative humidity 55 ± 10%, and a 12:12 h light–dark cycle, with *ad libitum* access to food and water. Animals were monitored daily for signs of distress (e.g., weight loss, reduced mobility, piloerection). No adverse events were observed. Humane endpoints were predefined but not reached. To ensure exposure consistency across experimental groups, both chambers were identical in size and airflow and received ambient air from the same external source under normobaric conditions. Environmental parameters were standardized across chambers, including temperature, humidity, and light–dark cycle. ${\mathrm{PM}}_{2.5}$ concentrations within the chambers were monitored daily, and outdoor levels were simultaneously recorded using a BAM 1020 monitor located approximately 200 m from the exposure site.

### 4.3. Air Analysis

${\mathrm{PM}}_{2.5}$ concentrations inside the chambers were monitored daily with a digital analyzer, and outside using a beta attenuation monitor BAM 1020 (Met One Instruments, Inc., Grant Pass, OR, USA; Beta Source: ^14^C (carbon–14), 60 µCi ± 15 µCi (2.22 MBq)), equipped with a photomultiplier tube beta detector and an organic plastic scintillator at a flow rate of 16.7 L min^−1^. Data are expressed in µg/m^3^ and provided by “Algoritmos y Mediciones Ambientales SpA” from the “Las Encinas Monitoring Station” located 200 m from the chambers, also available through the National Air Quality Information System. NO_2_ and CO concentrations were consistent across chambers, as the filtration system did not retain these gases.

### 4.4. Study Design

This study employed a two-generation exposure model to evaluate the cumulative effects of sustained ${\mathrm{PM}}_{2.5}$ exposure across developmental windows encompassing prenatal, postnatal, and juvenile life stages. The experimental design involved three generations (Figure 6): founder females (G0) were exposed from birth and produced the first filial generation (G1) through mating with unexposed males. G1 females, maintained under continuous exposure, subsequently generated the second filial generation (G2). Histological analysis was intentionally restricted to G2 females, which were sampled at postnatal day 82. This selection was based on the rationale that G2 animals experienced continuous exposure from early development through reproductive maturity, thereby representing a life-course exposure model prior to first pregnancy. Only G2 females were sampled at postnatal day 82, representing animals with lifelong multigenerational exposure integrating prenatal, postnatal, and juvenile/adult developmental periods. This multigenerational approach was selected to approximate real-world environmental scenarios in which individuals are exposed to persistent pollutants across successive developmental stages and potentially across generations. This multigenerational approach was selected to model human scenarios in which women may experience sustained environmental pollutant exposure across their own development and that of previous generations, potentially accumulating biological effects. However, although this design enhances ecological relevance, it does not allow definitive discrimination between cumulative multigenerational effects and single-generation adaptive responses. Therefore, the uterine phenotype observed in G2 should be interpreted as the integrated outcome of sustained exposure rather than as evidence of a strictly transgenerational mechanism. By sampling only G2 animals, we assessed the integrated impact of continuous exposure spanning two complete reproductive cycles, distinguishing this model from acute or single-generation designs that may not capture cumulative tissue-level adaptations. The sampling endpoint (postnatal day 82) corresponds to reproductive maturity after establishment of regular estrous cyclicity, ensuring all animals were at comparable developmental stages. All animals were monitored by vaginal cytology to confirm the presence of regular estrous cycles, and only nulliparous females exhibiting consistent cyclicity were included in the study. Only nulliparous females with regular estrous cycles, confirmed by daily vaginal cytology and at least two consecutive complete cycles, were included. Uterine samples were collected at a fixed chronological endpoint (postnatal day 82), after confirmation of reproductive maturity. Group allocation was stratified by body weight using a computer-generated sequence, and investigators responsible for histological and image analyses were blinded to group assignment. This exposure model has been previously characterized in both FA and NFA groups with respect to estrous cycle staging, circulating reproductive hormone levels, and baseline uterine histology under identical environmental conditions [6,37]. In that study, no significant differences in cycle distribution, endocrine profiles, or phase-associated histological architecture were detected between exposure groups. Nevertheless, tissue collection was not restricted to a specific estrous phase at the time of euthanasia. While prior characterization indicates that chronic ${\mathrm{PM}}_{2.5}$ exposure under these conditions does not disrupt cyclical uterine organization, residual phase-dependent variability cannot be completely excluded and should be considered when interpreting epithelial remodeling endpoints. For contextual purposes, an inhalation-based exposure index was calculated using a 22-day reference window corresponding to the approximate gestational length in rats, providing an order-of-magnitude comparison between chambers. Importantly, this calculation does not represent total lifetime exposure from conception to postnatal day 82, but rather a standardized short-term window intended to avoid overinterpretation of cumulative-dose estimates in the absence of animal-specific ventilation measurements and deposition modeling. All experimental procedures were conducted in accordance with Chilean Law 20.380 on animal protection and the Guide for the Care and Use of Laboratory Animals (2011), and were approved by the Scientific Ethics Committee of the Universidad de La Frontera (Approval No. 122/20; 17 November 2020). This study was conducted and reported in accordance with the ARRIVE 2.0 guidelines.

### 4.5. Exposure Groups

We used two groups of nulliparous adult female Sprague Dawley rats from the second generation (G2), representing animals with continuous transgenerational ${\mathrm{PM}}_{2.5}$ exposure from conception (Figure 6). All G2 animals were bred and housed under standardized laboratory conditions, including a 12:12 h light–dark cycle, constant temperature (22 ± 2 °C), and relative humidity (55 ± 10%), with *ad libitum* access to food and water. Animals were randomly selected from different litters, without prior genetic manipulation, to reduce potential confounding factors associated with lineage or selective breeding. Two exposure groups were formed as described in [7]. The filtered air (FA) group comprised 12 Sprague Dawley rats housed in exposure chambers in which ambient air was pre-filtered using high-efficiency particulate air (HEPA) filters to eliminate ${\mathrm{PM}}_{2.5}$ and other airborne pollutants, thereby serving as the control condition to assess uterine histoarchitecture under low-exposure baseline conditions (Figure 6). The non-filtered air (NFA) group consisted of 12 Sprague Dawley rats continuously exposed to unfiltered ambient air containing ${\mathrm{PM}}_{2.5}$ and combustion-derived particles, simulating an environment of chronic exposure to wood smoke pollution and allowing evaluation of real-world effects of airborne particulate matter on uterine histology. Animals were allocated to FA or NFA groups using a computer-generated randomization sequence concealed from investigators involved in histological processing and image analysis, implementing partial blinding to minimize detection bias. Sample size (n=12 per group) was defined based on effect sizes and group sizes reported in previous studies by our group, which consistently detected significant differences in uterine histological parameters under comparable environmental exposure conditions, and was estimated to provide sufficient statistical power (>80%) to detect moderate biological effects (d≈1.2) at an α level of 0.05 [7].

### 4.6. Immunohistochemistry

Consecutive paraffin-embedded sections of the uterine horn were cut at a thickness of 5 µm using a rotary microtome. Sections were deparaffinized in xylene (3 × 5 min) and rehydrated through a graded ethanol series (100%, 90%, 70%, and 50%; 5 min each). Primary antibodies and their corresponding dilutions are detailed in Supplementary Document S2, and all antibodies had been previously validated for use in uterine tissue. Antibodies were diluted in Tris/HCl buffer (pH 7.8) containing 8.4 mM sodium phosphate, 3.5 mM potassium phosphate, 120 mM NaCl, and 1% bovine serum albumin. Sections were incubated with primary antibodies overnight at 4 °C in a humidified chamber, after which immunodetection was performed using the IHC Select^®^ HRP/DAB kit (DAB150, Merck, Darmstadt, Germany) ) according to the manufacturer’s instructions. Stained sections were examined and imaged using a Leica^®^ DM750 light microscope equipped with a Leica^®^ MC170HD digital camera (Leica Microsystems GmbH, Wetzlar, Germany), and negative controls were performed by omission of the primary antibody, and no specific signal was detected under these conditions.

### 4.7. TUNEL Assay

Apoptotic cell death was evaluated using the TUNEL (Terminal deoxynucleotidyl transferase dUTP nick end labeling) assay. Paraffin-embedded uterine horn sections were cut at a thickness of 5 µm, deparaffinized in xylene (3 × 5 min), and rehydrated through a descending ethanol gradient (100%, 90%, 70%, and 50%; 5 min each). Detection of TUNEL-positive (TUNEL^+^) cells was performed using the ApopTag^®^ Plus Peroxidase In Situ Apoptosis Detection Kit (Code S7101, Merck, Darmstadt, Germany) according to the manufacturer’s instructions. Signal development was achieved using diaminobenzidine as chromogen, and nuclei were counterstained with methyl green.

### 4.8. Image Quantification

Immunohistochemistry images were processed using ImageJ software v1.45. The Color Deconvolution plugin [96] was employed to separate hematoxylin and DAB chromogens by channels and to determine the areas of positive staining. The percentage of positively stained area was calculated as the ratio of the DAB-positive area to the total tissue section area. Images included the three histological layers of the uterus, comprising the complete endometrial structure. Images obtained from TUNEL, Ki-67, and MGMT assays were analyzed using the StrataQuest software (v7.1.1.138). Analysis parameters were established based on nuclear detection using hematoxylin or methyl green staining as nuclear markers. Thresholds were kept constant across groups. Automated cell segmentation was performed through nuclear marker-based masking to accurately define nuclear boundaries. For all markers, only cells exhibiting nuclear DAB-positive staining were classified as positive, whereas any cytoplasmic or extracellular signal was excluded from the analysis. This approach ensured that the quantification accurately reflected nuclear labeling and marker-specific cellular activity. The number of positively labeled cells was quantified per uterine stratum: endometrium (ENDO), circular muscle layer (Myo-C), vascular layer (Myo-V), and longitudinal muscle layer (Myo-L). To calculate the labeling rate for TUNEL, Ki-67, and MGMT, the number of positive cells per stratum was normalized to 1000 total cells. This standardization enabled valid comparisons between exposure groups.

### 4.9. Immunofluorescence, Confocal Spectral Microscopy and Second Harmonic Generation (SHG)

Paraffin-embedded uterine horn sections were cut at a thickness of 7 µm, deparaffinized in xylene (3 × 5 min), and rehydrated through descending ethanol concentrations (100%, 90%, 70%, and 50%; 5 min each). Primary antibodies were diluted in Tris/HCl buffer (pH 7.8) containing 1% bovine serum albumin, and sections were incubated overnight at 20 °C in a humidified chamber. Nuclear counterstaining was performed using TO-PRO-3 iodide (1:1000; V–6630). Multichannel fluorescence and SHG images were acquired using a Zeiss LSM780 NLO spectral multiphoton microscope (Axio Observer.Z1; Carl Zeiss, Oberkochen, Germany) operated with the ZEN 2011 software (v8.0; Carl Zeiss). Fluorescence imaging was performed using a 405 nm excitation laser, and images were acquired with EC Plan–Neofluar 20× objective lenses (NA 0.5, working distance 2.0 mm) or a Plan–Apochromat 63× oil immersion objective (NA 1.4, working distance 0.19 mm). SHG imaging was performed using an EC Plan–Neofluar 40×/1.30 oil immersion objective to visualize endogenous collagen fibers, with SHG signals excited by a pulsed infrared laser (Chameleon; Coherent Inc., Santa Clara, CA, USA) at 790 nm and emission collected using a 388–427 nm band-pass filter.

### 4.10. Protocol Statement

A predefined experimental protocol, including the research question, study design, and analysis plan, was established prior to study initiation. However, this protocol was not formally registered in a public repository.

### 4.11. Statistical Analysis

All quantitative results are reported as mean ± standard deviation (SD). Data normality was evaluated with the D’Agostino–Pearson omnibus test, and homogeneity of variances between the two exposure groups was confirmed with an F-test (homoscedasticity assumption). When both assumptions were met, unpaired two-tailed Student’s *t*-tests were applied to compare group means. A *p*-value < 0.05 was considered statistically significant. Analyses were performed with GraphPad Prism 10.4.2 for macOS (GraphPad Software, San Diego, CA, USA), using a 95% confidence interval. The chosen tests and sample sizes satisfied the assumptions of parametric analysis and were aligned with the study design and primary endpoints. No data were excluded post hoc; all experimental units (*n* = 24) were retained, and no imputation of missing values was necessary.

## 5. Conclusions

Chronic exposure to ${\mathrm{PM}}_{2.5}$ derived from wood smoke during preconceptional stages is associated with significant alterations in uterine morphology in nulliparous rats, even before the onset of reproductive activity. In a context consistent with sustained tissue hypoxia, marked structural alterations were observed, characterized by ECM remodeling with increased and disorganized collagen fiber content, a pro-inflammatory environment marked by TNF-α overexpression, and increased expression of VEGF-A consistent with modulation of angiogenesis-related pathways. In parallel, alterations in cellular homeostasis and markers associated with genomic maintenance were identified, including reduced cell proliferation, a trend toward increased apoptosis, and decreased expression of the DNA repair-associated protein MGMT. These findings suggest that prolonged environmental exposure to biomass-derived pollutants may compromise the structural integrity and tissue-level adaptability of the uterus at early stages, even in the absence of changes in circulating hormone levels or classical markers of endometrial receptivity such as HB-EGF. The lack of detectable endocrine disruption supports the hypothesis of a predominantly local effect on the uterine microenvironment, independent of the hypothalamic–pituitary–gonadal axis. Taken together, these results provide preclinical evidence of adverse uterine alterations associated with wood smoke-derived ${\mathrm{PM}}_{2.5}$ exposure and underscore the need for future research aimed at clarifying its potential implications for fertility-related processes, implantation competence, and pregnancy outcomes under conditions of chronic air pollution exposure. 

## Data Availability

The complete dataset supporting the findings of this study is available in the Zenodo repository https://doi.org/10.5281/zenodo.16798095 under the Creative Commons Attribution 4.0 International license.

## Supplementary Materials

The following supporting information can be downloaded at: https://www.mdpi.com/article/10.3390/ijms27104289/s1.

## Abbreviations

The following abbreviations are used in this manuscript:

BAM	Beta attenuation monitor
CO	Carbon monoxide
COL I	Collagen type I
COL III	Collagen type III
COL IV	Collagen type IV
CV	Coefficient of variation
DAB	3,3^′^-Diaminobenzidine
DNA	Deoxyribonucleic acid
ECM	Extracellular matrix
ENDO	Endometrium
FA	Filtered air
FGFR-1	Fibroblast growth factor receptor 1
FLT-1	Fms-related tyrosine kinase 1
G0	Founder generation
G1	First filial generation
G2	Second filial generation
HB-EGF	Heparin-binding epidermal growth factor-like growth factor
HEPA	High-efficiency particulate air
HIF-1α	Hypoxia-inducible factor 1 alpha
IHC	Immunohistochemistry
IF	Immunofluorescence
IVF	In vitro fertilization
KDR-1	Kinase insert domain receptor 1
MGMT	O^6^-Methylguanine-DNA methyltransferase
MMP	Matrix metalloproteinase
Myo-C	Circular muscle layer
Myo-L	Longitudinal muscle layer
Myo-V	Vascular layer
NFA	Non-filtered air
NO_2_	Nitrogen dioxide
PM	Particulate matter
${\mathrm{PM}}_{2.5}$	Particulate matter with aerodynamic diameter ≤2.5 µm
${\mathrm{PM}}_{10}$	Particulate matter with aerodynamic diameter ≤10 µm
ROS	Reactive oxygen species
SD	Standard deviation
SHG	Second harmonic generation
TGF-β	Transforming growth factor beta
TIMP	Tissue inhibitor of metalloproteinases
TNF-α	Tumor necrosis factor alpha
TUNEL	Terminal deoxynucleotidyl transferase dUTP nick end labeling
VEGF-A	Vascular endothelial growth factor A
WHO	World Health Organization
