# Supplementary material for: Uterine Vulnerability to Environmental PM2.5: Chronic Wood Smoke Exposure Alters Morphogenesis Before First Pregnancy
Source: Int J Mol Sci. 2026 May 12;27(10):4289. doi: 10.3390/ijms27104289 (PMC13207024; doi:10.3390/ijms27104289)
Supplement: Supplementary file 1 [file ijms-27-04289-s001.zip › Supplementary Document 1.pdf]

SUPPLEMENTARY DOCUMENTS

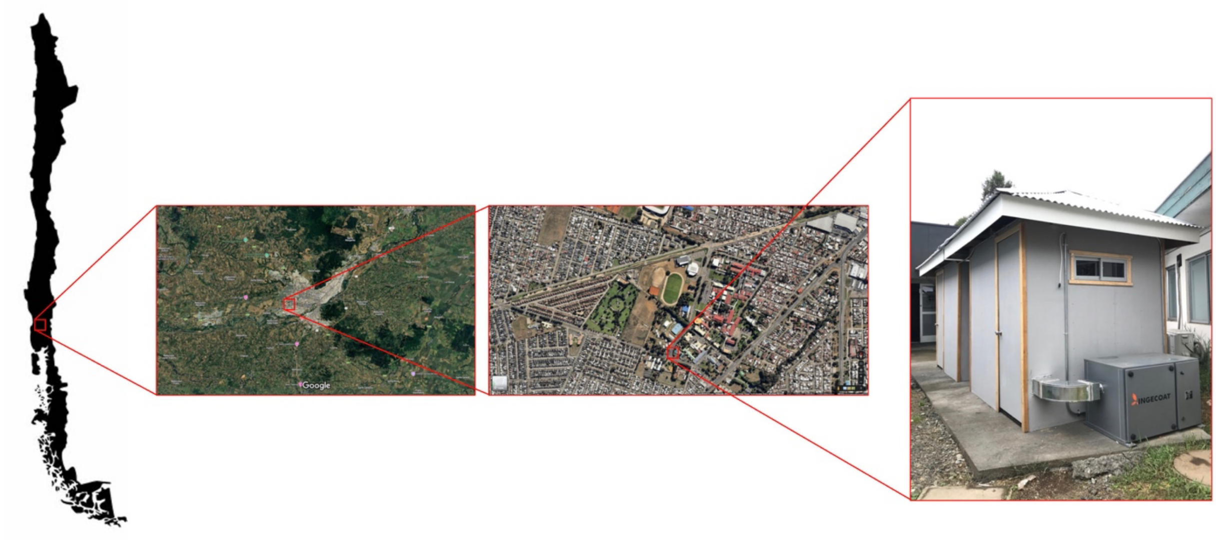

Supplementary Document 1. Exposure chambers in the courtyard of the Faculty of Medicine at the University of La Frontera. located in the downtown area of Temuco. 500 meters away from the environmental air monitoring station (-38.7496844990132. -72.6188400896599).
