# Supplementary material for: Uterine Vulnerability to Environmental PM2.5: Chronic Wood Smoke Exposure Alters Morphogenesis Before First Pregnancy
Source: Int J Mol Sci. 2026 May 12;27(10):4289. doi: 10.3390/ijms27104289 (PMC13207024; doi:10.3390/ijms27104289)
Supplement: Supplementary file 1 [file ijms-27-04289-s001.zip › Supplementary Document 2.pdf]

SUPPLEMENTARY DOCUMENTS

Supplementary Document 2. Primary antibodies used for immunohistochemistry (IHC) and immunofluorescence (IF) in uterine tissue. List of antibodies employed in this study. including antibody name. product code, technique applied (IHC or IF), working dilution, and supplier.

Antibody	Code	Technique	Dilution	mManufacter
Anti-Col I	NB600-408	IHQ	1:200	Novus
Anti-Col III	NB600-594	IHQ	1:300	Novus
Anti-Col IV	NB120-6586	IHQ	1:300	Novus
Anti-TGF-B	NBP2-45137	IHQ, IF	1:100/1:500	Novus
Anti-TNF-A	MBS438099	IHQ, IF	1:200/1:500	Mybiosource
Anti-HB-EGF	SC-365182		1:200	Santa Cruz
Anti-HIF1-A	SC13515	IHQ, IF	1:100/1:100	Santa Cruz
Anti-VEGF-A	AB231260	IHQ	1:200/1:100	Abcam
Anti-Kdr-1	AB2349	IHQ	1:50	Abcam
Anti-Flt-1	AB2350	IHQ	1:50	Abcam
Anti- FGFR-1	NB6001287	IHQ	1:100	Novus
Anti-Ki67	NB110-89719	IHQ	1:200	Novus
Anti-MGMT	MBS2026596	IHQ	1:200	Mybiosource
Anti-Vimentina	AB5733	IF	1:100	Abcam
Anti-Factor de Von Willebrand	F-3520	IF	1:250	Thermo Fisher
