# Supplementary material for: Uterine Vulnerability to Environmental PM2.5: Chronic Wood Smoke Exposure Alters Morphogenesis Before First Pregnancy
Source: Int J Mol Sci. 2026 May 12;27(10):4289. doi: 10.3390/ijms27104289 (PMC13207024; doi:10.3390/ijms27104289)
Supplement: Supplementary file 1 [file ijms-27-04289-s001.zip › Supplementary Document 3.pdf]

SUPPLEMENTARY DOCUMENTS

Supplementary Document 3. Averages of particulate matter (MP) and CO recordings during the study period (June 15 - September 30, 2021; southern hemisphere) and annually.

	Study period	Annually
PM2.5 [ug/m^3]	48.8 ± 36.1 (CV: 74.0%)	26.2 ± 30.6 (CV: 117%)
MP10 [ug/m^3]	56.9 ± 38.3 (CV: 67.3%)	36.6 ± 30.8 (CV: 84.2%)
CO [ppm]	0.78 ± 0.49 (CV: 61.5%)	0.44 ±0.43 (CV: 97.7%)
