# Supplementary material for: Uterine Vulnerability to Environmental PM2.5: Chronic Wood Smoke Exposure Alters Morphogenesis Before First Pregnancy
Source: Int J Mol Sci. 2026 May 12;27(10):4289. doi: 10.3390/ijms27104289 (PMC13207024; doi:10.3390/ijms27104289)
Supplement: Supplementary file 1 [file ijms-27-04289-s001.zip › Supplementary Document 4.pdf]

SUPPLEMENTARY DOCUMENTS

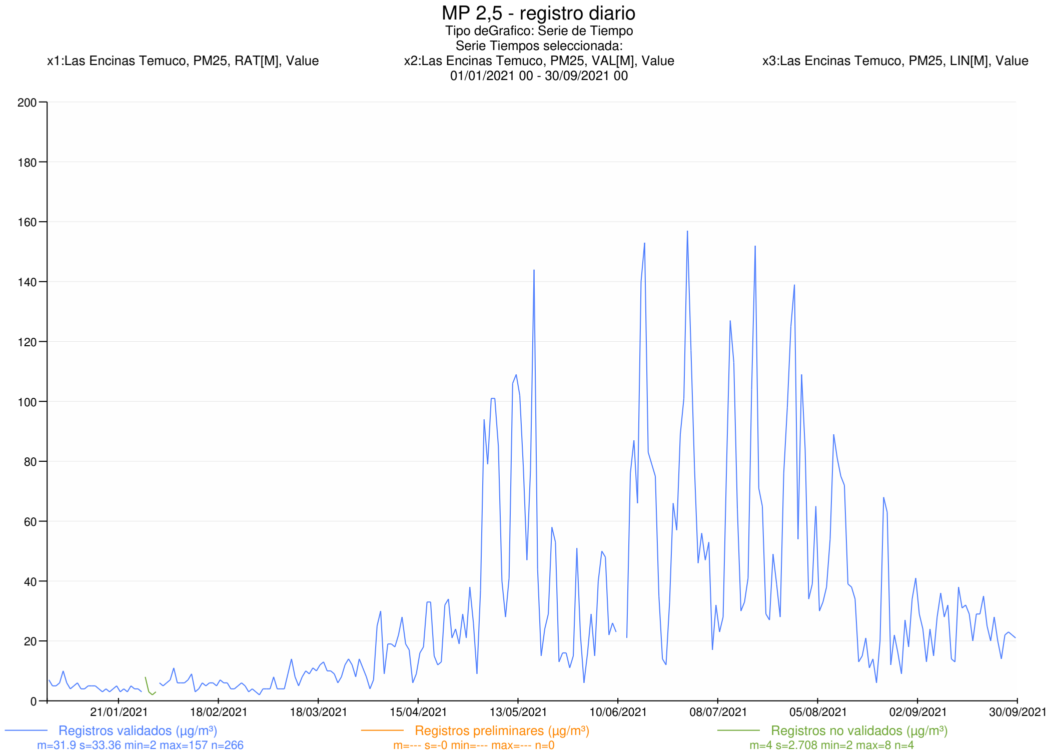

Supplementary Document 4. Figure III. Daily PM2.5 concentrations (µg/m³) recorded at the “Las Encinas Monitoring Station” (Temuco, Chile) during the study period (January 1 to September 30, 2021). PM2.5 levels were measured externally using a beta attenuation monitor BAM 1020 (Met One Instruments, Inc., Grant Pass, OR, USA), equipped with a carbon-14 (60 µCi ±15 µCi) beta source and a photomultiplier tube beta detector with an organic plastic scintillator, operating at a flow rate of 16.7 L/min. Data were provided by “Algoritmos y Mediciones Ambientales SpA” and accessed via the National Air Quality Information System (https://sinca.mma.gob.cl). The chart differentiates between validated (blue), preliminary (orange), and unvalidated (green) records.
