# Supplementary material for: Uterine Vulnerability to Environmental PM2.5: Chronic Wood Smoke Exposure Alters Morphogenesis Before First Pregnancy
Source: Int J Mol Sci. 2026 May 12;27(10):4289. doi: 10.3390/ijms27104289 (PMC13207024; doi:10.3390/ijms27104289)
Supplement: Supplementary file 1 [file ijms-27-04289-s001.zip › Supplementary Document 5.pdf]

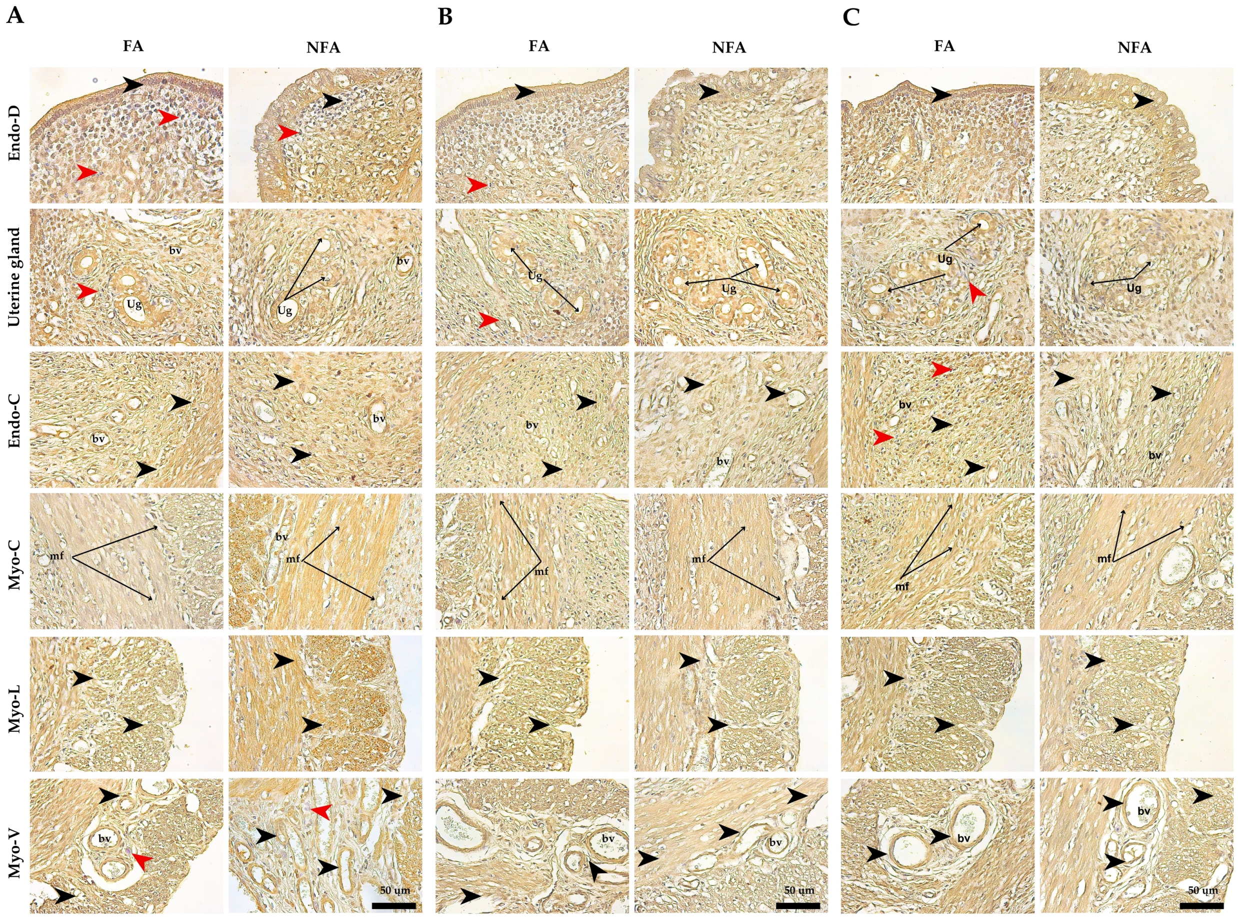

Supplementary Document 5. Immunohistochemical distribution of collagen types I (panel A), III (panel B) and IV (panel C) in transverse sections of uterine horns from nulliparous Sprague-Dawley rats (82 days old) exposed to filtered air (FA) or to wood-smoke $PM_{2.5}$ (non-filtered air, NFA). Representative photomicrographs are shown for the deep endometrial layer (DL), uterine glands (Ug), compact endometrial layer (CL), circular myometrial layer (CML), longitudinal myometrial layer (LML) and vascular layer (VL). Black arrowheads denote collagen-positive cells or fibres; red arrowheads indicate weak/negative staining. Blood vessels (bv) and smooth-muscle fibres (mf) are labelled where appropriate. Compared with FA, NFA samples exhibit a broader and more intense collagen signal across endometrial epithelium, stroma and both myometrial layers, corroborating the quantitative increase in area fraction for COL I ($p = 0.0171$), COL III ($p = 0.0213$) and COL IV ($p = 0.0283$) reported in Fig. 2D–F. Scale bars = 50 μm.
