# Supplementary material for: Uterine Vulnerability to Environmental PM2.5: Chronic Wood Smoke Exposure Alters Morphogenesis Before First Pregnancy
Source: Int J Mol Sci. 2026 May 12;27(10):4289. doi: 10.3390/ijms27104289 (PMC13207024; doi:10.3390/ijms27104289)
Supplement: Supplementary file 1 [file ijms-27-04289-s001.zip › Supplementary Document 6.pdf]

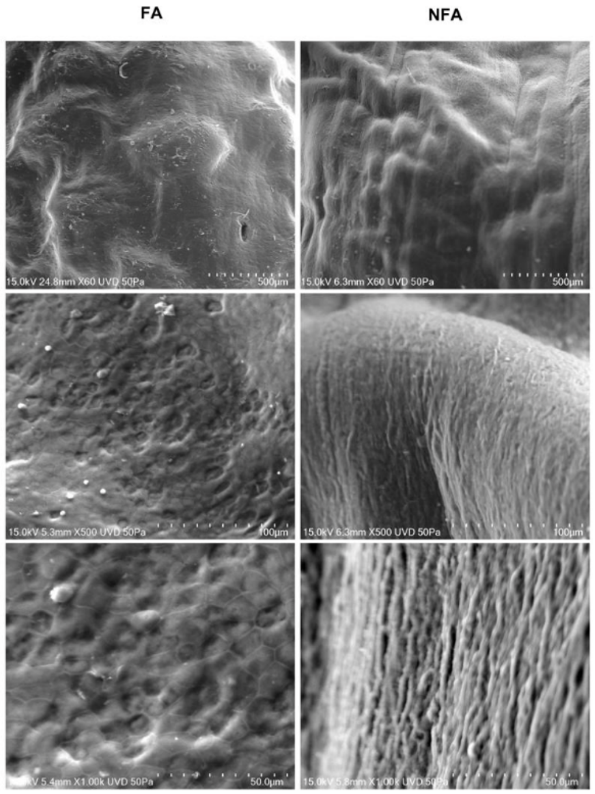

Supplementary Document 6. *Scanning electron microscopy (SEM) of the luminal epithelium of uterine horns from 82-day-old nulliparous Sprague-Dawley rats continuously exposed to filtered air (FA, left) or wood-smoke PM2.5 (non-filtered air, NFA, right). (a–c) Low-magnification view (60 ×; scale bar = 500 µm) shows an intact epithelial sheet in both groups; however, NFA specimens display parallel folds and fibrillar surface projections, contrasting with the "cobblestone" arrangement of cuboidal cells in FA. (d–e) At 500 × (scale bar = 100 µm) the NFA surface exhibits thicker, longitudinal micro-ridges suggestive of fibrillar reorganization, whereas FA maintains uniformly short microvilli. (f–g) High-magnification view (1,000 ×; scale bar = 50 µm) highlights the density and alignment of apical protrusions: isolated microvilli dominate in FA, while an interlaced filamentous network is evident in NFA, giving a "fibrous mesh" appearance. These ultrastructural alterations parallel the nonsignificant reduction in HB-EGF expression (p = 0.1275) and indicate that chronic PM2.5 exposure subtly remodels the endometrial surface architecture even in the absence of marked changes in this receptivity marker.*
