# Supplementary material for: Uterine Vulnerability to Environmental PM2.5: Chronic Wood Smoke Exposure Alters Morphogenesis Before First Pregnancy
Source: Int J Mol Sci. 2026 May 12;27(10):4289. doi: 10.3390/ijms27104289 (PMC13207024; doi:10.3390/ijms27104289)
Supplement: Supplementary file 1 [file ijms-27-04289-s001.zip › Supplementary Document 7.pdf]

SUPPLEMENTARY DOCUMENTS

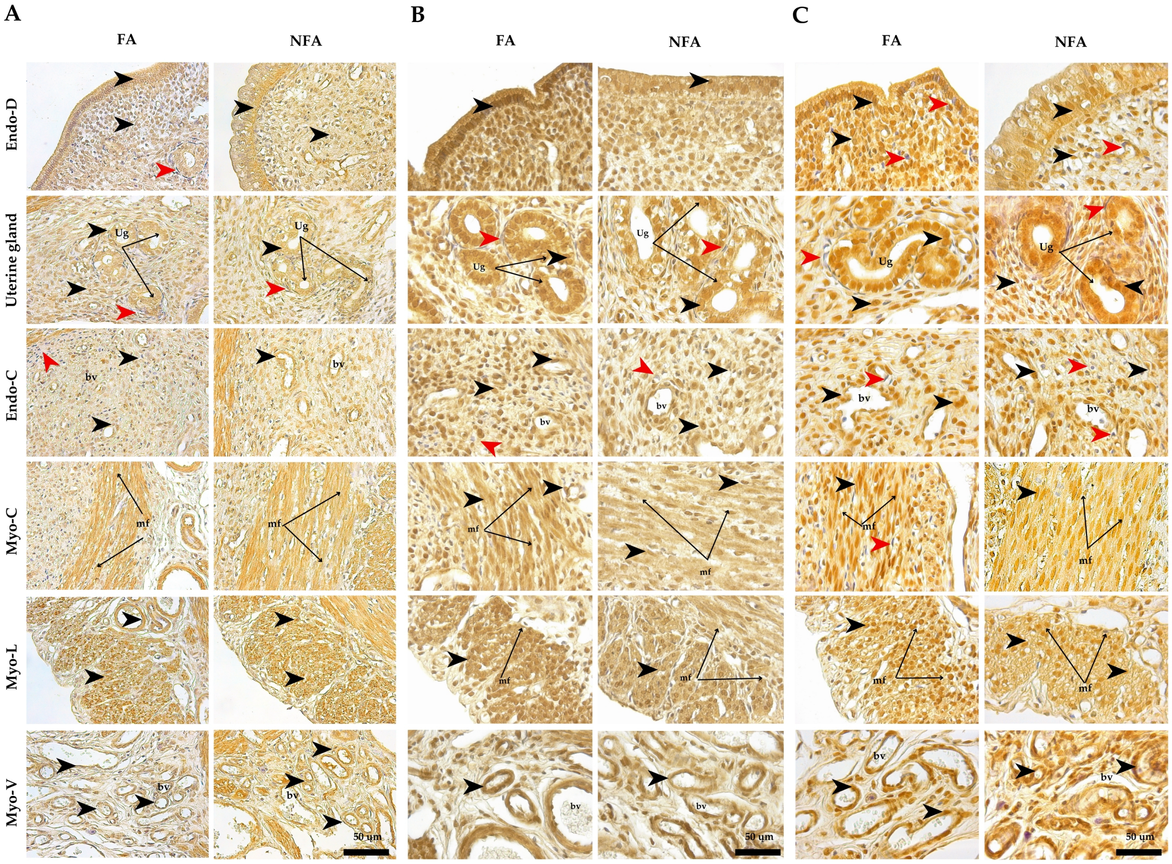

Supplementary Document 7. Immunohistochemical profiling of the FGFR/FLT-1/KDR-1 angiogenic axis in uterine horns of nulliparous rats chronically exposed to wood-smoke PM2.5. Transverse sections from 82-day-old Sprague-Dawley rats housed in filtered air (FA) or non-filtered air (NFA) are shown. Panel A (FGFR): NFA animals exhibit a broader and more intense FGFR signal throughout endometrial and myometrial compartments, consistent with hypoxia-driven angiogenic activation. Panel B (FLT-1): PM2.5 exposure does not alter FLT-1 receptor distribution or staining intensity relative to FA. Panel C (KDR-1): KDR-1 receptor expression likewise remains comparable between exposure groups. Layers: DL, deep endometrial layer; Ug, uterine glands; CL, compact endometrial layer; CML, circular myometrium; VL, vascular layer; LML, longitudinal myometrium. Black arrowheads = immunopositive cells/fibres; red arrowheads = immunonegative cells; bv = blood vessels; mf = muscle fibres. Scale bars = 50 µm.
