# Supplementary material for: Uterine Vulnerability to Environmental PM2.5: Chronic Wood Smoke Exposure Alters Morphogenesis Before First Pregnancy
Source: Int J Mol Sci. 2026 May 12;27(10):4289. doi: 10.3390/ijms27104289 (PMC13207024; doi:10.3390/ijms27104289)
Supplement: Supplementary file 1 [file ijms-27-04289-s001.zip › Supplementary Document 8.pdf]

SUPPLEMENTARY DOCUMENTS

Supplementary Document 8. Quantification of uterine markers associated with hypoxia, inflammation, extracellular matrix remodeling, angiogenesis, cell proliferation, apoptosis, and DNA repair in nulliparous rats exposed to filtered air (FA) or non-filtered air (NFA). Immunohistochemical quantification of stained area (%) is shown for hypoxia (HIF1-α), inflammation (TGF-β, TNF-α), collagen subtypes (COL I, III, IV), and angiogenic/growth factors (VEGF-A, FLT-1, KDR-1, FGFR-1, HB-EGF). Cell proliferation (Ki67+), apoptosis (TUNEL+), and DNA repair (MGMT+) were quantified as the number of positive cells per 1000 total cells in total uterine tissue and individual strata: endometrium (Endo), circular muscle layer (Myo-C), vascular layer (Myo-V), and longitudinal muscle layer (Myo-L). Data are presented as mean ± standard deviation for each experimental group (Student's t-test; $p < 0.05$).

		**FA**	**NFA**	**p-value**
Hypoxia	HIF1-α	25,43±3.10	30.54 ±6.97	0.0037
Inflammation [% stained area]	TGF-β	24.43±4.00	20.47±3.18	0.0009
	TNF-α	23.67±5.55	28.33±4.72	0.0024
Collagen [% *stained area*]	COL I	35.99 ± 6.58	44.97 ± 8.20	0.0171
	COL III	46.62 ± 12.72	56.75 ± 9.45	0.0213
	COL IV	35.78 ± 10.52	46.93 ± 12.66	0.0283
Growth factors and uterine angiogenesis [% *stained area*]	VEGF-A	22.57 ± 6.74	31.70 ± 8.63	<0.0001
	FLT-1	24.49 ± 2.88	22.32 ± 5.79	0.0897
	KDR-1	25.89 ± 9.19	29.39 ± 6.87	0.1524
	FGFR-1	24. 46 ± 7.61	36.23 ± 7.51	0.5133
	HB-EGF	43.68 ± 9.82	38.90 ± 3.14	0.1275
KI67+/1000 cells	Total	772 ± 125.30	443 ± 110.8	0.0001
	Endo	813 ± 74.46	647 ± 138.8	0.019
	Myo-C	872 ± 69.6	377 ± 182.5	0.0012
	Myo-V	731 ± 184.9	495 ± 108.00	0.0223
	Myo-L	876 ± 147.8	253 ± 93.28	0.0001
TUNEL+/1000 cells	Total	636 ± 57.71	705 ± 27.99	0.0442
	Endo	598 ± 65.74	690 ± 92.62	0.1083

	Myo-C	665 ± 92.15	704 ± 87.30	0.5094
	Myo-V	660 ± 163.30	667 ± 134.6	0.9495
	Myo-L	872 ± 51.22	762 ± 187.10	0.2374
MGMT+ /1000 cells	Total	524 ± 248.00	199 ± 128.0	0.0303
	Endo	466 ± 298.0	288 ± 183.0	0.2868
	Myo-C	562 ±264.6	144 ± 118.9	0.0120
	Myo-V	564 ± 184.8	204 ± 122.2	0.0066
	Myo-L	509 ± 266.1	160 ± 118.7	0.0281

Endo: endometrium; Myo-C: circular muscle layer; Myo-V: vascular layer; Myo-L: longitudinal muscle layer. Values are expressed as mean ± SD. Percent stained area applies to immunohistochemistry (% area). and cell counts represent positive cells per 1000 total cells.
